# *Fusobacterium* spp. target human CEACAM1 via the trimeric autotransporter adhesin CbpF

**DOI:** 10.1080/20002297.2018.1565043

**Published:** 2019-01-24

**Authors:** Matthew L. Brewer, David Dymock, R. Leo Brady, Bernhard B. Singer, Mumtaz Virji, Darryl J. Hill

**Affiliations:** a School of Biochemistry, University of Bristol, UK; b School of Oral and Dental Sciences, University of Bristol, UK; c Institut für Anatomie, Universitätsklinikum Essen, Essen, Germany; d School of Cellular & Molecular Medicine, University of Bristol, UK

**Keywords:** *Fusobacterium*, CEACAM1, CEA, host–pathogen interaction, adhesion, binding, trimeric autotransporter adhesin, TAA, Type V secretion, *Fusobacterium nucleatum*

## Abstract

*Neisseria meningitidis, Haemophilus influenzae*, and *Moraxella catarrhalis* are pathogenic bacteria adapted to reside on human respiratory mucosal epithelia. One common feature of these species is their ability to target members of the carcinoembryonic antigen-related cell adhesion molecule (CEACAM) family, especially CEACAM1, which is achieved via structurally distinct ligands expressed by each species. Beside respiratory epithelial cells, cells at the dentogingival junction express high levels of CEACAM1. It is possible that bacterial species resident within the oral cavity also utilise CEACAM1 for colonisation and invasion of gingival tissues. From a screen of 59 isolates from the human oral cavity representing 49 bacterial species, we identified strains from *Fusobacterium* bound to CEACAM1. Of the *Fusobacterium* species tested, the CEACAM1-binding property was exhibited by *F. nucleatum* (Fn) and *F. vincentii* (Fv) but not *F. polymorphum* (Fp) or *F. animalis* (Fa) strains tested. These studies identified that CEACAM adhesion was mediated using a trimeric autotransporter adhesin (TAA) for which no function has thus far been defined. We therefore propose the name CEACAM binding protein of *Fusobacterium* (CbpF). CbpF was identified to be present in the majority of unspeciated *Fusobacterium* isolates confirming a subset of *Fusobacterium* spp. are able to target human CEACAM1.

## Introduction

The mucosal pathogens *Neisseria meningitidis* (meningococci, Nm), *Haemophilus influenzae* (Hi), and *Moraxella catarrhalis* (Mx) are human-specific organisms commonly resident in the nasopharynx of healthy individuals. However, for reasons still not fully known, Hi and Mx can cause a number of localised infections including sinusitis, otitis media, and exacerbations of chronic obstructive pulmonary disease (COPD). In addition, Nm and occasionally Hi and Mx may disseminate from the nasopharynx to cause serious infections such as septicaemia and meningitis [1–4].

Studies of potential targets on host cells for adhesion have led to the discovery that antigenically distinct adhesins of these three species are able to target members of the human carcinoembryonic antigen-related cell adhesion molecules (CEACAMs [5–7]). The meningococcal and related *N. gonorrhoeae* (Ng) CEACAM-binding ligands, the Opa proteins, have been studied extensively [7–10]. In the case of Hi, the outer membrane proteins P5 and P1, with β-barrel structures, have been shown to bind to the receptors [6,11,12]. However, in the case of Mx, the CEACAM-binding ligands are the ubiquitous surface proteins A1 (UspA1) and A2V (UspA2V), a trimeric autotransporter adhesin (TAA) [5,13,14]. The term autotransporter was initially used to describe the soluble IgA protease from Ng [15]. All autotransporters, a protein superfamily of Gram-negative bacteria, share the common features of an N-terminal signal sequence and a C-terminal β-barrel forming domain, which facilitates passage of the passenger domain across the outer membrane [16]. Unlike the monomeric-secreted autotransporters such as IgA proteases, the passenger domain of trimeric autotransporters often remain attached to the surface of the bacterial cells where they perform diverse adhesive functions (reviewed in [17]). TAAs were first proposed to be a subfamily of autotransporters [18] but are now considered to be a distinct protein family of the autotransporter superfamily [17]. Despite the initial nomenclature of autotransporter continuing to be used, we now know a number of other proteins have roles to play in the surface presentation of such proteins [reviewed in 19].

The CEACAM family belongs to the Immunoglobulin superfamily and include epithelial and polymorphonuclear cell-expressed members such as CEACAM1, CEACAM3, CEA, CEACAM6, and CEACAM8 whose distribution in tissues and functions may be divergent [20,21]. Of the cell surface-expressed members of the family, CEACAM1 (previously known as BGP and CD66a) has the broadest tissue distribution and is expressed on the apical surfaces of epithelial cells of human mucosa, cells of myeloid lineage as well as on some endothelial cells [20–22]. Focussing specifically on oral/respiratory tissues, CEACAM expression on normal epithelial cells in oral, tonsillar, and lung tissues has been reported [22–24]. We have demonstrated the expression of the receptor on the apical surfaces of tonsillar epithelium [25], where the receptor may be available for microbial colonisation. Since increased receptor density demonstrably increases the chances of cellular invasion by bacteria [26], these observations suggest that CEACAMs may play a critical role in mucosal colonisation and pathogenesis. CEACAM1, CEA, and CEACAM6 are expressed in human junctional epithelium [27]. However, whether other oro-respiratory bacterial colonisers/pathogens besides Nm, Hi, and Mx target CEACAMs has not been fully investigated.

In this study, we undertook a survey of oral bacteria and from a screen of oral isolates comprising 20 genera and at least 51 individual species of bacteria, we identified that strains belonging to the *Fusobacterium* genus, Gram-negative anaerobic bacteria, were capable of binding to CEACAM1. Further, we show that two distinct species, *F. nucleatum* (Fn) and *F. vincentii* (Fv), as well as several unspeciated clinical isolates of *Fusobacterium* bind to CEACAM1. Historically, Fn and Fv were members of the same species (*F. nucleatum*), however, recent studies have since shown the distinction between the species based on whole-genome similarity [28,29]. The interaction of *Fusobacterium* spp. with CEACAM1 is mediated by a trimeric autotransporter which we have named CbpF. Identification of surface proteins of *Fusobacterium* involved in pathogenesis may ultimately lead to novel therapeutic strategies to eliminate the numerous diseases caused by these bacteria.

## Materials and methods

### Bacterial strains and culture

A comprehensive list of bacterial species and strains used in this study is provided (Tables S1 and S2). The collection consisted of clinical isolates and type strains as detailed. The control isolates of *N. meningitidis* (C751) and *M. catarrhalis* (MX1) used in the study have been described previously [5,10].

To culture oral species, fastidious anaerobe agar (supplied by LabM, Heywood, UK) supplemented with 10% horse blood was inoculated and grown under anaerobic conditions (N_2_:CO_2_:H_2_ 8:1:1 in a Don Whitley MkIII Anaerobic Cabinet, Bingley, UK) for 2–3 days at 37°C. Bacteria were subcultured into fastidious anaerobe broth media (LabM), and in the case of the *Prevotella, Porphyromonas, Flavobacterium, Bacteroides, and Campylobacter* species, the medium was supplemented with hemin (5 μg ml^−1^), and grown under the same conditions.

### Antibodies

Polyclonal antiserum against a 15-mer peptide (KW15) corresponding to residues 255–269 of Fn CbpF (KNDYKDANDIDVNKW) was raised in rabbits using standard protocols. Anti-polyhistidine mouse monoclonal antibody was purchased from Qiagen (Manchester, UK) and used at 0.2 μg ml^−1^. YTH71.3 was purchased from AbDSerotec (Oxford, UK) and Kat4c was purchased from Dako UK Ltd, Cheadle, UK.

### Preparation of bacterial lysates

Bacterial cells were harvested by centrifugation (12,000 *g*, 15 min). The pellet was resuspended in phosphate buffered saline (PBS) containing protease inhibitor cocktail [1 mM phenylmethylsulphonyl fluoride (PMSF), 1 µM E-64, 1 µM pepstatin A, 6 nM bestatin, and 100 µM EDTA; PIC]. Bacterial suspensions were lysed by freeze-thaw, aliquots were solubilised in SDS-NaOH, and their optical densities were measured at 260 nm to allow standardisation as described previously [30].

### Immunodotblotting

Soluble CEACAM1-Fc constructs and control CD33-Fc constructs used have been described previously [5,10,31]. For analysis of CEACAM binding by immunodotblotting, equivalent amounts of bacterial lysate were immobilised on to nitrocellulose membranes. Non-specific binding sites were blocked with 3% bovine serum albumin (BSA) in PBS containing 0.05% Tween 20 (PBST; 1 h, Room Temperature (RT)). The membranes were then overlaid with either CEACAM1-Fc, CEACAM1 N-domain construct (N-Fc), CEACAM1 I91A mutant construct, or CD33-Fc negative binding control for 1 h, RT (1–2 μg ml^−1^ as indicated in 1% BSA-PBST). Blots were washed three times (PBST containing 0.9% w/v NaCl) and overlaid with anti-human-Fc antibody conjugated to alkaline phosphatase (AP) in 1% BSA-PBST (1 h, RT). Following washing, the binding of the receptor–antibody complex to bacterial lysates was detected by incubation with nitroblue tetrazolium (NBT) and 5 bromo-4 chloro-3-indolyl phosphate (BCIP) in AP buffer (100 mM Tris-base, 100 mM NaCl, 5 mM MgCl_2_). The relative levels of receptor binding were determined by densitometric analysis using the NIH Scion Image program.

### SDS-PAGE and western blotting of Fusobacterial lysates

Fusobacterial lysates were subjected to sodium dodecyl sulphate–polyacrylamide gel electrophoresis (SDS-PAGE, 5–10%) under reducing conditions (180 V, up to 1 h, RT). Gels were either stained with Coomassie brilliant blue or the proteins transferred to nitrocellulose or PVDF membranes (300 mA, 1 h, 4°C). Non-specific binding sites were blocked using 3% BSA in PBST (1 h, RT). The membranes were overlaid with CEACAM1-Fc or control constructs (1 µg ml^−1^) and receptor binding was detected as described in immunodotblotting.

### Immunoprecipitation of the Fusobacterial ligand with CEACAM1 N-Fc

To capture the bacterial CEACAM-binding ligand, 100 µl of protein A (50% slurry) coupled to sepharose CL-4B (Sigma, Gillingham, UK) was incubated with either 50 µg of N-Fc or the control construct CD33-Fc overnight at 4°C and subsequently washed three times with PBSB (Dulbecco’s complete PBS, Fisher Scientific, Loughborough, UK) to remove any unbound receptor. Simultaneously, overnight cultures of bacteria were suspended in 100 mM octyl-β-d-glucopyranoside (OG; final concentration) in PBSB containing PIC. Samples were mixed end-over-end overnight at 4°C. Insoluble bacterial material was removed by centrifugation at 10,000 *g* for 15 min. Soluble supernatant was divided in two 500 μl aliquots and incubated with either N-Fc or CD33-Fc receptor–Protein A sepharose complexes for 3 h at 4°C. After washing three times with 50 mM OG and PBSB, samples were dissociated at 100°C for 10 min in SDS-PAGE dissociation buffer and analysed by electrophoresis and Western blotting.

### Identification of CEACAM-binding ligand of *Fusobacterium nucleatum* by MALDI-TOF and N-terminal sequencing

The area of the gel corresponding to the CEACAM-binding ligand identified by Western blotting was excised and subjected to in-gel trypsin digestion. The resulting peptides were analysed by matrix-assisted laser desorption ionisation time-of-flight (MALDI-TOF) mass spectrometry, carried out by the University of Bristol Proteomics Facility (http://www.bristol.ac.uk/biochemistry/proteomics/). The spectra of the tryptic digestion products were acquired using a PE Biosystems Voyager-DE STR MALDI-TOF mass spectrometer. Peak lists were analysed using PROFOUND peptide-mapping program (Rockerfeller University).

Following transfer to PVDF membrane, proteins were detected with Coomassie Brilliant blue and the ligand band excised and subjected to Edman degradation to determine the N-terminal sequence, carried out by the University of Bristol Proteomics Facility.

### Treatment of Fusobacterial lysates with formic acid

As several trimeric autotransporters have been shown to readily dissociate in formic acid [32], whole-cell lysates of *F. nucleatum* were incubated overnight in 70% (v/v) formic acid at RT in the dark. Samples were subsequently freeze-dried to remove the volatile formic acid and resuspended in sample buffer prior to electrophoresis and Western blotting. Western blots were probed with soluble CEACAM1-Fc and control constructs as described above.

### PCR and sequencing

Genomic DNA was extracted from Fn isolates using a DNeasy Tissue Kit (Qiagen) according to the manufacturer’s instructions. The *cbpF* genes were amplified using CbpF primers For1/Rev1 (Table S3) and purified using a PCR cleanup kit (Qiagen). Genes were sequenced through the DNA sequencing service (http://www.dnaseq.co.uk), using appropriate forward and reverse primers to ensure coverage (Table S3). Sequence analysis and alignment were performed using Lasergene EditSeq, SeqBuilder, and MegAlign softwares. Sequence data have been submitted to the GenBank database under accession numbers JQ922431–JQ922439.

### Generation of recombinant CbpF

CbpF primers For3/Rev2 were used to amplify the *cbpF* gene encoding amino acids 7–395 of the mature protein from clinical isolate 2B3 and cloned into pQE30UA (Qiagen) according to the manufacturer’s instructions. Additionally, the pOPINE vector was used for the generation of recombinant CbpF from Fn ATCC 25586 (group I; CbpF primers For5/Rev5) and from the clinical strain 2B3 (group II; CbpF primers For6/Rev6). Ligation-independent cloning (LIC) was employed for creating the pOPINE-based plasmid using the In-Fusion® cloning kit (Clontech, Saint-Germain-en-Laye, France). Primers for these constructs are detailed in Table S3.

The pQE30-based plasmids were transformed into *E. coli* strain M15 previously transformed with pREP4 regulation plasmid according to the manufacturer’s instructions (Qiagen). The pOPINE-based expression vectors were transformed into Rosetta™ (DE3) pLacI cells (Novagen, WI, USA). M15 transformants were grown in the presence of 100 μg ml^−1^ ampicillin and 25 μg ml^−1^ kanamycin. Rosetta™ cells were grown in the presence of 100 μg ml^−1^ ampicillin and 34 μg ml^−1^ chloramphenicol.

A 10-ml LB broth culture containing the appropriate antibiotics was inoculated with the transformed expression strain and grown overnight at 37°C. Five-hundred millilitres LB broth containing the relevant antibiotics then was inoculated using the overnight culture and grown to late log phase (~OD_600_ 1.8). In order to induce protein expression, isopropyl thiogalactopyranoside (IPTG) was added to a final concentration of 1 mM. Following 1 h induction, bacteria were collected by centrifugation (6,000 *g* for 15 min) and pellets stored at −80°C for subsequent protein purification. Bacterial pellets were thawed and 5 ml lysis buffer was added per gram wet weight of pellet (lysis buffer 50 mM NaH_2_PO_4_, 300 mM NaCl, 10 mM imidazole). Bacteria were lysed by sonication on ice and insoluble material was collected by centrifugation at 10,000 *g* for 30 min at 4°C. Recombinant proteins were purified from the supernatant by binding to Ni-NTA resin columns (Qiagen) washed with wash buffer (lysis buffer containing 100 mM imidazole) and eluted with elution buffer (lysis buffer containing 250 mM imidazole). Protein-containing fractions were exhaustively dialysed against PBS, assayed for protein content using the Pierce BCA protein assay (Fisher Scientific, Loughborough, UK), and stored at 4°C for further analysis.

### ELISAs

ELISA plates (96 well, Dynex, Chantilly, USA) were coated with recombinant CbpF (rCbpF; 3 pmol) in carbonate buffer pH 9.5 by overnight incubation at 4°C. Wells were blocked with 3% w/v BSA (diluted in PBS-T; PBS with 0.05% v/v TWEEN® 20, Sigma, Gillingham, UK) for 1 h. Triplicate wells were incubated with the relevant CEACAM-Fc construct diluted in 1% BSA (w/v in PBS-T) for 1 h at RT, where the final concentrations are detailed where required. Following a minimum of three washes with PBS-T, the AP-conjugated anti-IgG Fc secondary antibody was then incubated for a further hour before three more wash steps. Plates were developed using SigmaFast™ p-Nitrophenyl phosphate substrate (Sigma) and absorbance was measured at 405 nm (A405). Data underwent background subtraction and standardisation for graphical presentation.

### CEACAM-Fc generation

CEA-Fc and mouse CEACAM1b-Fc were purchased from Sino Biological, Wayne, USA. CEACAM1-Fc, CEACAM3-Fc, CEACAM6-Fc, CEACAM8-Fc, CEACAM1 ΔN-Fc, and N-Fc were constructed and purified using methods as previously described [33]. The CEACAM1 mutants were constructed using site-directed mutagenesis from a CEACAM1-3 (containing N-A1-B1 domains) construct within the pINFUSE2 vector (NEB; shuttle vector to create Fc fusion proteins). The vectors were sequenced to confirm the mutation and transiently transfected into COS1 cells before purifying protein using Protein A affinity chromatography. Primers for these constructs are detailed in Table S4.

## Results

### *Fusobacterium nucleatum*-CEACAM binding identification from a panel of oral pathogens

**Figure 1.**
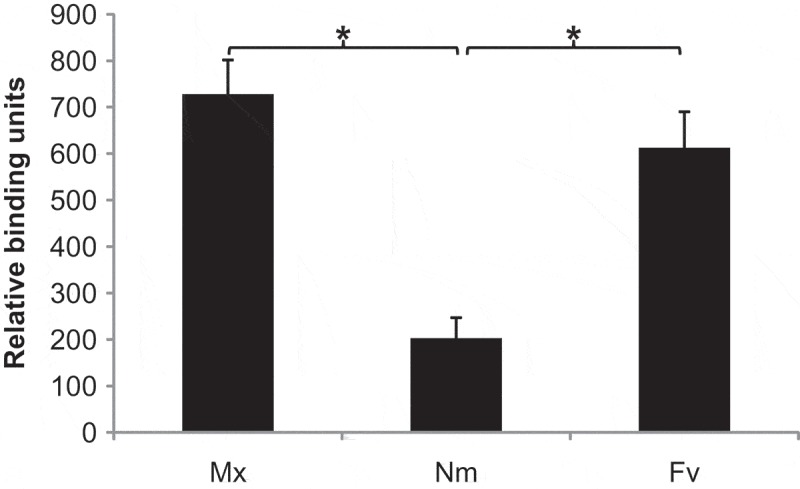
Densitometric plot of CEACAM1-Fc binding to oral bacterial species. Bacterial isolates were standardised by spectrophotometry, applied to nitrocellulose, and overlaid with CEACAM1-Fc (1 μg ml^−1^). Mx: CEACAM-binding UspA2V expressing *M. catarrhalis* strain MX1 (positive control), Nm: Opa negative variant *N. meningitidis* strain C751 (negative control), Fnv: *Fusobacterium vincentii*. Means and standard errors of six independent experiments are shown. *p < 0.001.

A range of bacterial species associated with the human oral cavity (Table S1) were screened for CEACAM1 binding in receptor overlay experiments. Bacterial lysates were standardised as described in Experimental Procedures and equal amounts were dotted onto nitrocellulose membranes. Samples were overlaid with CEACAM1-Fc, comprising all extracellular CEACAM1 domains fused to the human immunoglobulin G (IgG) Fc domain (or CD33-Fc used as a control), and binding subsequently detected using anti-Fc secondary antibodies conjugated to AP. Of the isolates screened initially, the strongest positive CEACAM1 binding was displayed by the obligate anaerobe *Fusobacterium vincentii* (Fv; Figure 1; Data showing comparable detection of CEACAM1-Fc and CD33-Fc as well as representative immunodotblot data are shown in Figure S1).

### The nature of CEACAM-binding by F. nucleatum

**Figure 2.**
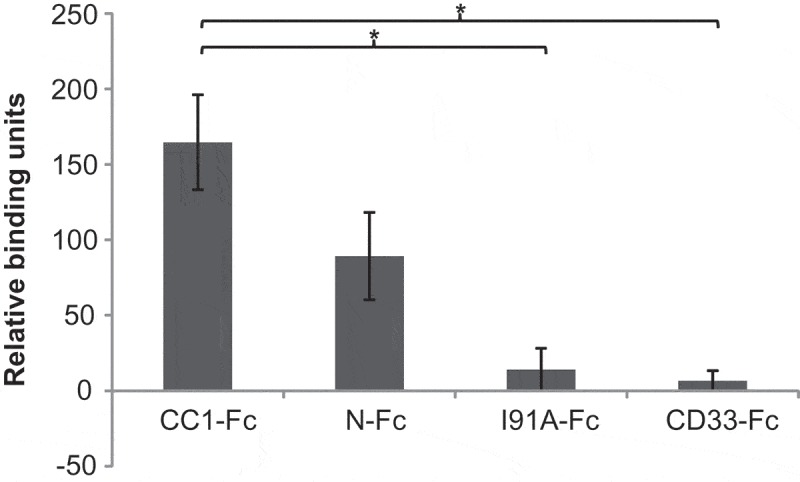
CEACAM1 N-domain binding to *F. vincentii*. Densitometric plot of Fnv overlaid with CEACAM1 (CC1-Fc), CEACAM1 N-domain (N-Fc), CEACAM1 I91A construct (I91A-Fc), or CD33 construct (CD33-Fc) used as a negative control. All constructs were overlaid at 2 μg ml^−1^. Mean values and standard errors of three independent experiments are shown. *p < 0.05.

*N. meningitidis, H. influenzae*, and *M. catarrhalis*, all target CEACAM1 via antigenically variable ligands, however, all are capable of binding via the IgV-like N-domain of CEACAM1. To elucidate whether this was the case for *Fusobacterium*, bacterial lysates dotted onto nitrocellulose were overlaid with a chimeric construct comprising CEACAM1 N-domain and human IgG Fc (N-Fc). Binding of the N-Fc construct to Fv was observed confirming that, in common with the ligands of other respiratory pathogens, the N-domain of CEACAM1 alone is capable of supporting Fv binding (Figure 2; Representative immunodotblot data and Fc construct comparisons are shown in Figure S2). Previous studies have identified a common overlapping region on the CFG face of CEACAM1 N-domain which supports the interaction of diverse ligands from Mx, Nm, Ng, and Hi strains and involves isoleucine at position 91 in each case. On substitution of isoleucine 91 with alanine at this face of CEACAM1, binding of these four bacterial species was abrogated [10,34,35]. Interestingly, Fv was not able to bind to the I91A CEACAM1-Fc mutant construct indicating the same or overlapping region on CEACAM1 is also targeted by fusobacteria (Figure 2). No binding of the CD33-Fc control protein was observed.

### Identification of the of *Fusobacterium* CEACAM1-binding ligand

**Figure 3.**
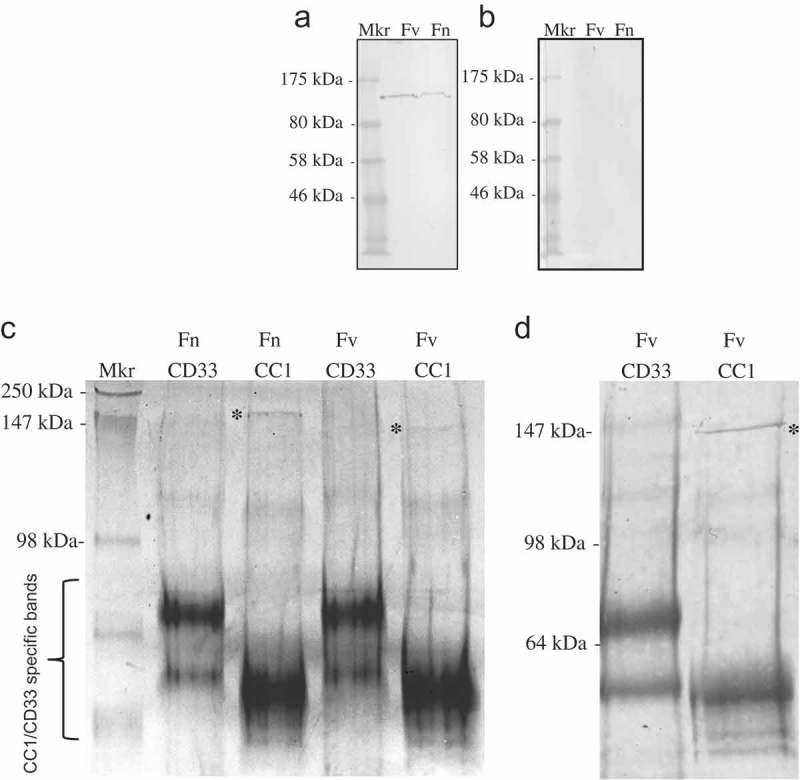
Identification of the CEACAM-binding ligand of *F. nucleatum and F. vincentii* following Western blotting and co-immunoprecipitation. Western blot of Fv (a) of Fnn (b) under non-reducing conditions overlaid with CEACAM1-Fc (lane 1) or CD33-Fc (lane 2; both 1 μg ml^−1^) and detected via an appropriate secondary antibody conjugated to AP. CEACAM1 binding to a band of ~150 kDa was observed to be present in lane 1 but no binding was observed in the CD33-Fc control (lane 2). (c) Coomassie stained gel showing co-precipitated proteins from octyl glucoside extracts of Fn and Fv using CD33-Fc or CEACAM-Fc as indicated. A band of ~150 kDa was co-precipitated with CEACAM1-Fc but not CD33-Fc for each subspecies as indicated (*). (d) Corresponding Coomassie stained Western blot of Fv co-precipitated using either CD33 or CEACAM1-Fc constructs. The band indicated (*) represents the CEACAM1-binding ligand of Fv and was subsequently subjected to N-terminal sequencing. Representative data from one of three repeated experiments are shown.

Whole cell Fv lysate was subjected to SDS-PAGE and corresponding Western blots overlaid with CEACAM1-Fc. A high molecular weight protein of Fv can be observed binding to CEACAM1 at ~150 kDa that was not present in controls that used CD33-Fc (Figure 3(a)). In addition to Fv, a second species (*nucleatum*; Fn) was also shown to bind to CEACAM1-Fc in Western blot overlay (Figure 3(b)), suggesting the ability to target CEACAMs is not restricted to members of the *vincentii* species.

In order to identify the CEACAM1-binding ligand, type strains of *F. vincentii* ATCC 49256 and in addition *F. nucleatum* ATCC 25586 were used in co-precipitation studies as complete genome sequences are available for both these strains aiding peptide searches in the NCBI databases for the purposes of protein matching.

Co-precipitation of the *Fusobacterium* ligand with the receptor was carried out using the N- domain of CEACAM1 (N-Fc) construct (~50 kDa) due to similar size of the full-length CEACAM1-Fc and the bacterial ligand (~150 kDa). Co-precipitation with N-Fc yielded a protein of ~150 kDa not only from Fv, but also from Fn (Figure 3(c)). It is worth noting that the co-precipitated ligand(s) were eluted and separated under reducing conditions yet retain the molecular weight of ~150 kDa, suggesting either the ligand was a single protein or a stable multimer not containing cysteine residues. In addition, the ligand of Fn appears slightly larger than that of Fv indicative of some sequence variation between these proteins. No CEACAM-binding protein was observed in controls where CD33-Fc was used in place of N-Fc (Figure 3(c)). The protein co-precipitated with CEACAM1-Fc was subjected to in-gel tryptic digestion and subsequent analysis by MALDI-TOF mass spectrometry. This proved unsuccessful with a paucity of peptides released by the in-gel digestion even when the amount of starting material was regarded as sufficient.

### N-terminal sequencing of the CEACAM1-binding ligand of *F. nucleatum* and *F. vincentii*

CEACAM1 N-Fc co-precipitated protein from both Fv (Figure 3(d)) and Fn (not shown) were blotted onto polyvinylidene fluoride (PVDF) membrane, stained with Coomassie and Sequenced by Edman degradation. For Fn, the sequence of AAPVIKAGTAT was returned which corresponded to the Fn protein FN1499 (accession number AAL93625). For Fv, a different N-terminal sequence was returned (VPVIQGG) and corresponded to the Fv protein FNV1729 (accession number EAA24952). Despite possessing dissimilar N-terminal sequences, the two proteins are 88.8% identical over the whole gene products (Figure S3). Due to the lack of defined function, no nomenclature has been previously described for these proteins, which we have referred to as CEACAM binding proteins of *Fusobacterium* (CbpF).

### Properties of the CEACAM-binding protein of *Fusobacterium*, CbpF

CbpF of Fn and Fv are 479 and 471 amino acid residues in length, respectively. Both proteins possess similar 24 residue signal sequences (identified by SignalP; http://www.cbs.dtu.dk/services/SignalP/; version 3.0) yielding mature proteins with a predicted molecular mass of 48,209 Da for Fn and 47,348 Da for Fv. The disparity between the size of the CEACAM-binding ligand on Western blots (~150 kDa) and mature CbpF (~50 kDa) is therefore suggestive of a trimeric molecule. The slightly larger size of FN1499 fits well with the observed sizes of Fn and Fv ligands following CEACAM co-precipitation (Figure 3(b)). BLAST analysis of CbpF identified conserved domains typically found within the TAA family of proteins. In addition to CbpF, the CEACAM-binding ligand of *M. catarrhalis*, UspA1 is also a trimeric autotransporter protein forming a stable trimeric coiled region within which the CEACAM-binding domain is located [36]. Besides matching FN1499 and FNV1729 by BLAST as expected, three other fusobacterial proteins were identified with homology to CbpF within type strains namely FNP_1391 (accession numberEDK89173.1, *F. polymorphum*, 644 amino acids, E = 9e-68), FN0735 (accession number NP_603632, Fn, 617 amino acids E = 2e-65), and FN0471 (accession number NP_603368, Fn, 340 amino acids, E = 4e-58). Identities of all five fusobacterial proteins are shown in Figure S4. Whilst the proteins identified to bind CEACAM1 share the closest similarity, the presence of other homologues suggest that multiple variants of this trimeric autotransporter are present in *Fusobacterium* species.

**Figure 4.**
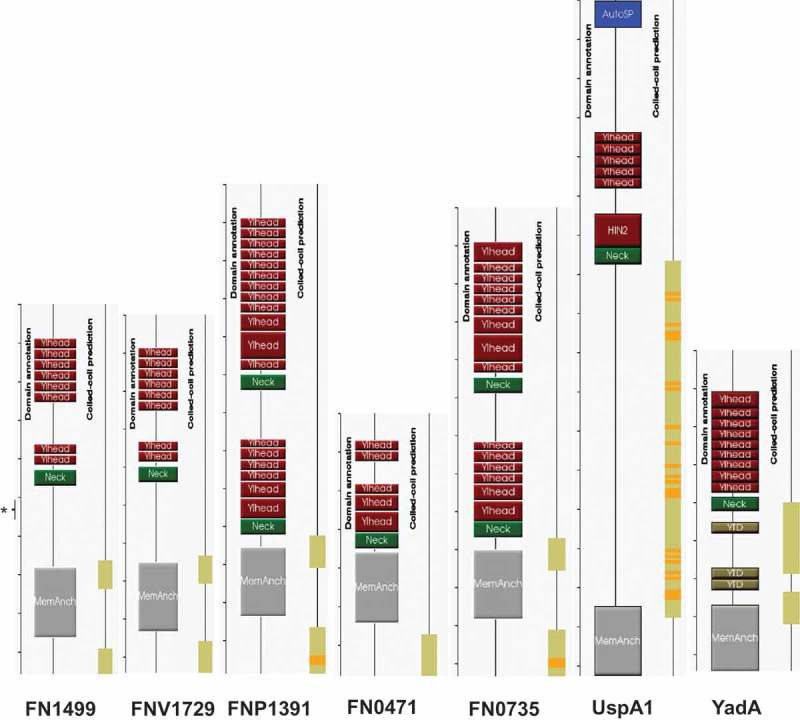
Identities and domain organisation of CbpF-like autotransporters from *Fusobacterial* spp. Domain annotation models of the family members indicating number of YadA-like head unit repeats and stalk coiled-coil regions relative to another CEACAM-binding autotransporter UspA1. Models of both UspA1 and YadA are included for comparative purposes. The approximate location of the KW15 epitope is indicated in FN1499 (*).

Using a search engine designed to identify and annotate domains within trimeric autotransporter adhesins [daTAA; 37], graphical representations of the domain arrangement of CbpF homologues were generated and are shown in Figure 4.

Full details of the TAA domains annotated are provided in the reference describing daTAA [37]. Domains pertinent to CbpF of Fn and Fv include YadA-like head domains, a left handed β-helix present in a number of trimetric autotransporters, including *Yersinia* spp. The domain possesses a highly conserved, relatively hydrophobic, SSAFG-like sequence likely to form the internal surface of a trimeric β-roll head. A neck region appears to link the head to a stalk lacking extensive coiled coil (in the case of TAAs three strands of α-helix which further coil around each other) observed in other TAAs such as UspA1 of *M. catarrhalis* but possesses short regions flanking the predicted β-barrel membrane anchor. Graphical representations of YadA and UspA1 are also shown for comparative purposes (Figure 4). A coiled-coil search using MARCOIL [38] also showed no evidence for the presence of coiled coils in the predicted stalk region.

### Immunological relatedness of Fv and Fn CbpFs and evidence for their trimeric nature

**Figure 5.**
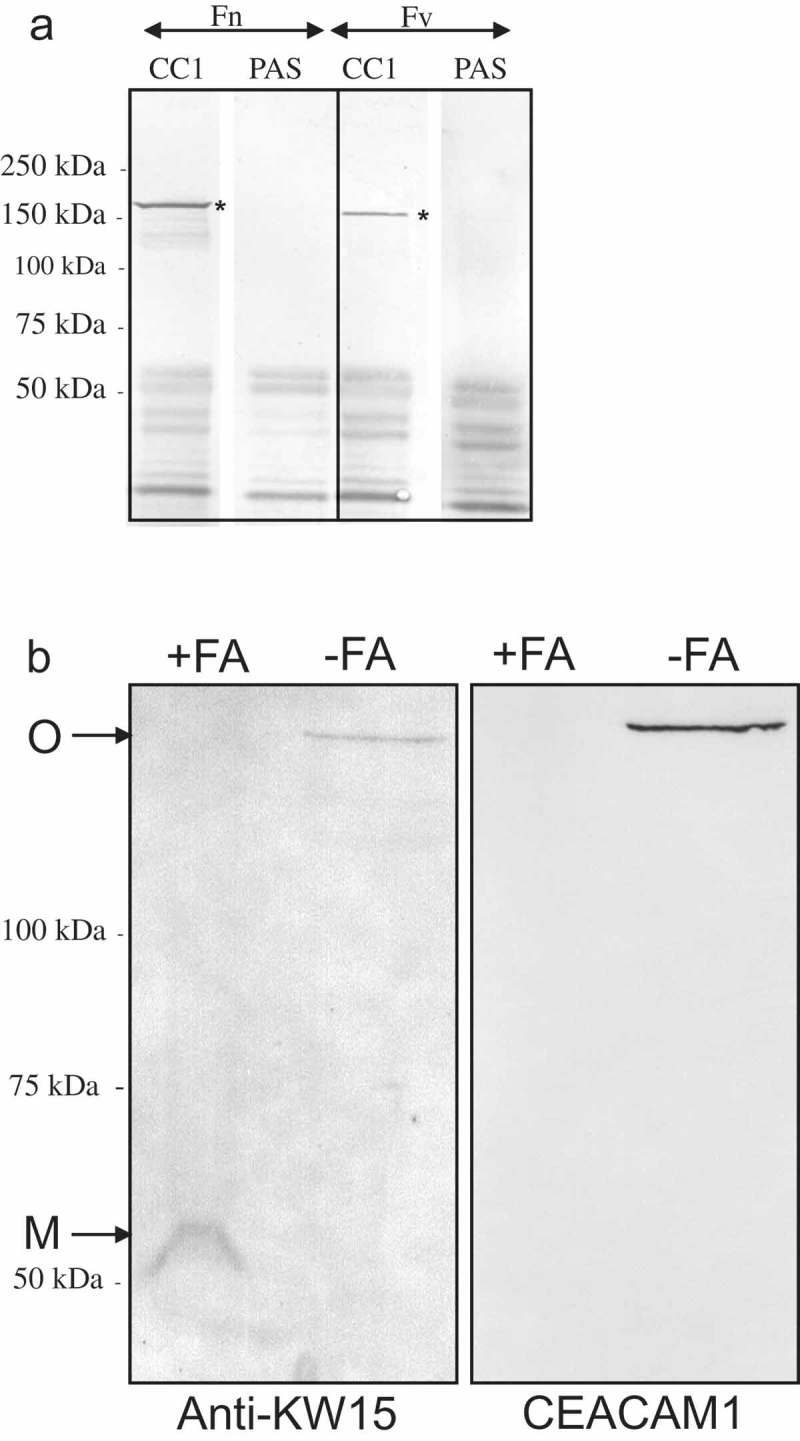
Immunological reactivity of anti-KW15 antiserum with co-immunoprecipitated proteins and proteins solubilised using formic acid. (a) Western blot showing binding of anti-KW15 antiserum to the protein co-precipitated using CEACAM1 (CC1) compared to the control co-precipitation which used protein A-sepharose (PAS) alone. Bands were observed at ~150 kDa (*) for both Fn and Fv as indicated. (b) Fn lysate treated with or without formic acid (FA) prior to electrophoresis and Western blotting. The blots were overlaid with anti-KW15 antiserum. Anti-KW15 bound to both a 150 kDa oligomeric (O) band and a 50 kDa monomeric protein band (M), whilst CEACAM1 only bound to the oligomeric form of CbpF. Data are representative of two independent experiments.

In order to validate that FN1499/FNV1479 represented CbpF, rabbit polyclonal antiserum was raised against a 15-mer peptide corresponding to residues 255–269 of Fn CbpF KNDYKDANDIDVNKW (KW15). This peptide was also conserved in CbpF of Fv representing residues 240–254. Anti-KW15 serum was bound to the co-precipitated CEACAM ligand of Fn as well as Fv (Figure 5(a)). Although the CEACAM-binding ligand of *M. catarrhalis* (UspA1) is a stable trimer, it can be dissociated into apparent monomers by heating for >5 min in SDS-sample dissociation buffer. This appears not to be the case for CbpF, unlike other autotransporters such as UspA1 and BadA, we did not observe ladder-like partial denaturation during electrophoresis [14,39]. In order to try to dissociate CbpF into monomeric subunits, whole cell lysates of Fn were solubilised in 70% formic acid which we have successfully used for other autotransporters previously [40]. Following lyophilisation to remove formic acid, samples were run on SDS-PAGE and corresponding Western blots overlaid with anti-KW15 and CEACAM1-Fc (Figure 5(b)). Following treatment with 70% formic acid, a band of ~50 kDa can be seen binding anti-KW15 compared the oligomeric form of the protein in the absence of formic acid. In contrast to the antiserum, CEACAM1 only binds to oligomeric CbpF and not the monomers generated by formic acid treatment. This suggested the region for CEACAM-binding was dependent on a folded protein conformation.

**Figure 6.**
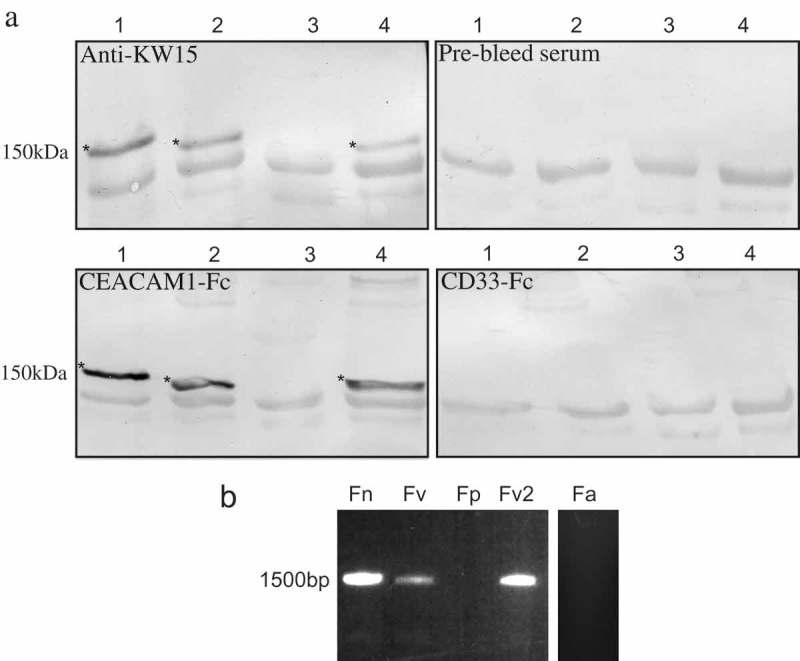
Comparison of CbpF presence in *Fusobacterium* species. (a) Western blots of lysates of *Fusobacterium* species *nucleatum* (Fn lane 1), *vincentii* (Fv lane 2), *polymorphum* (Fp lane 3), and a second *vincentii* strain (formerly *F. nucleatum* subspecies *fusiforme* (Fv2 lane 4)) were overlaid with anti-KW15, control pre-bleed serum, CEACAM1-Fc, and CD33-Fc as indicated. Note the CEACAM1-Fc and anti-KW15 binding bands in Fn, Fv, and Fv2 (*) but not in Fp. (b) PCR of *cbpF* from *F*. spp. Note the ~1,500 bp product for Fn, Fv, and Fv2 but no product as expected for Fp or Fa. Data are representative of two independent experiments.

In order to examine the presence of CbpF in other species of *Fusobacterium*, Western blots of lysates from Fn, Fv, Fp, and also a second Fv strain (ATCC51190) (Fv2; historically called *F. nucleatum* subsp. *fusiforme*) were overlaid with either CEACAM1-Fc or anti-KW15. The migration of anti-KW15 binding proteins was identical to that of the CEACAM-binding proteins of Fn, Fv, and Fv2, however, no anti-KW15 or CEACAM-binding was observed for Fp (Figure 6(a)), indicating that, in addition to Fn and Fv, an identical CbpF is present in Fv2. Further, no CEACAM1 or anti-KW15 binding was observed to the *F. animalis*-type strain, NCTC12276 (Fa; not shown). The presence of the *cbpF* gene was assayed by PCR using template DNA from Fn, Fv, Fp, Fv2, and Fa (Figure 6(b)). Products were observed of the expected size for Fn, Fv, and Fv2, however, no product was seen for Fp or Fa. Thus Fn, Fv, and Fv2 were shown to possess a CEACAM1-binding CbpF by PCR and proteomic analysis in contrast to Fp and Fa. Analyses of CbpF have been confined to immunodotblot and Western blot in this study, however surface expression of CbpF by Fv was confirmed using anti-KW15 (Figure S5).

### Presence and sequence similarity of CbpF in clinical isolates of *Fusobacterium spp*

**Figure 7.**
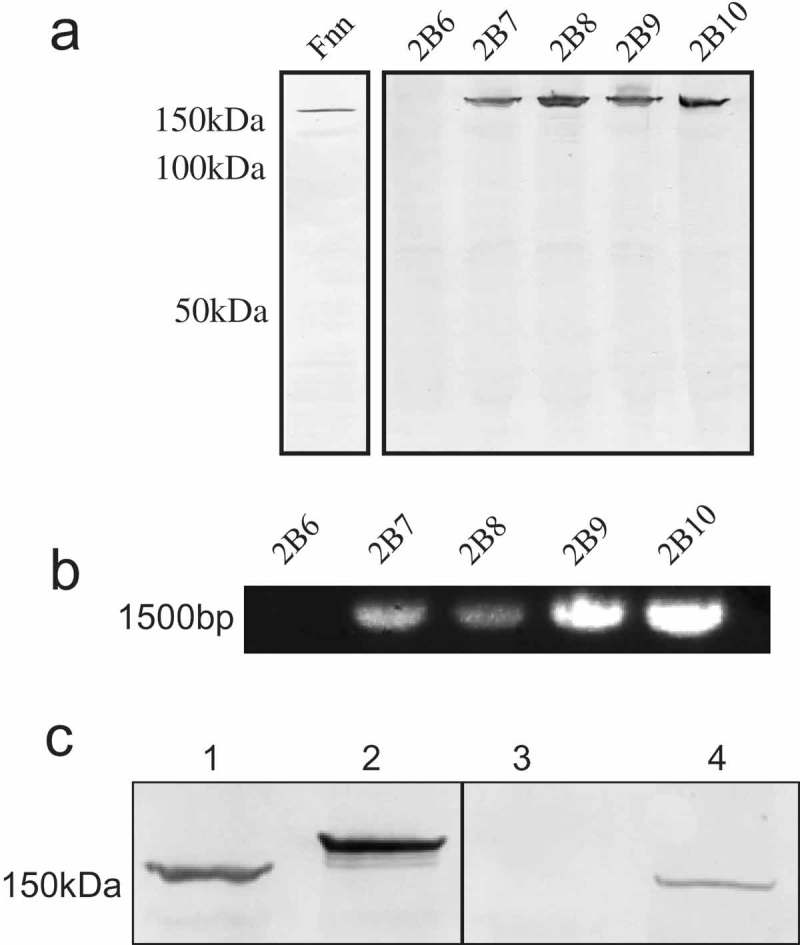
Analyses of CEACAM1 binding and *cbpF* gene presence in clinical isolates of *F. nucleatum*. (a) Western blot of representative clinical Fn isolates overlaid with CEACAM1-Fc. Strain designations are indicated above each lane. Note the absence of binding for 2B6 compared to the other isolates which expressed a ~150 kDa CEACAM1-binding protein. (b) PCR for *cbpF* from representative clinical isolates. Strain designations are indicated above each lane. Note the lack of product for isolate 2B6 corresponding with a lack of CEACAM1 binding observed in A. (c) Analysis of CbpF migration from the two distinct phylogenetic groups. Western blot overlay indicating the relative migration of CbpF from group I (lane 1 and 4) and group II (lane 2). Lane 1, 2B17, lane 2 2B16, lane 3 2B14 (non-binding control), and lane 4 2B13. Blot was overlaid with CEACAM1-Fc at 1 μg ml^−1^ and detected via an alkaline phosphatase-conjugated secondary antibody. Data are representative of two independent experiments.

Data suggest that CbpF is present in at least two of the five historical Fn subspecies. In order to determine the extent of CbpF expression in *Fusobacterium*, a panel of clinical isolates of mostly unspeciated *Fusobacterium* spp. (Figure 7(a); Table S2) was screened for CEACAM1 binding. Of the unspeciated *Fusobacterium* isolates, 18 out of the 31 strains examined (58.1%; see Figure 7(a) for examples) bound to CEACAM1 whilst 13 isolates displayed no CEACAM1 binding. In addition, the presence of the *cbpF* gene was examined by PCR (Figure 7(b)). The majority of strains that displayed CEACAM1 binding produced a gene product of ~1,500 bp by PCR. Of the non-binding strains, the majority lacked a gene product of appropriate size by PCR suggesting the gene may be absent. Hence, these isolates may be more akin to Fp or Fa. However, two non-binding isolates did give an appropriately sized PCR product (2B24 and 2B28, not shown), but the reasons for these isolates not binding to CEACAM1 remain to be elucidated. Indeed, not all strains of a species may express a protein for a number of reasons including phase variation which has been suggested for other autotransporters of *Fusobacterium* spp. [41]. Neither strain identified as *Fusobacterium* spp. (2B31 and 2B35) bound CEACAM1, nor did *F. periodonticum* (2B36). These findings highlight the presence of *cbpF* and its gene product CbpF are specifically within some, but not all, *Fusobacterium* species.

### Sequencing of CbpF from clinical isolates

In order to compare sequences of CbpF from clinical isolates, a number of strains were selected and PCR products were generated using the primers A and B (see experimental procedures). Sequences were obtained for strains 2B2, 3, 4, 8, 9, 13, 16, 17, and 32 (sequence alignments obtained together with Fn and Fv CbpF are shown in Figure S6). Overall sequence similarities suggest two main groups of related sequences with group I including 2B13, 2B17, 2B32, Fn, and Fv (87.7–99.8% sequence identity). Group II comprises 2B2, 2B3, 2B4, 2B8, 2B9, and 2B16 (90.3–100% sequence identity; Figure S7). Between the groups, the sequence identity varies between 77.0 and 82.1%. Whilst differences do exist throughout the sequence, the main difference between the two groups appears to be in the number of YadA-like head domains within CbpF, with group II possessing a greater number (at least 14) compared with group I (at least 8). Overall, group II CbpF members are larger than Group I CbpF by ~45 amino acids. Such differences in size were detectable in a CEACAM1 overlay by Western blot in which group II CbpF migrated at a higher molecular mass than group I CbpF (Figure 7(c)). The difference in sequence between group I and group II was validated using LC MS/MS. No identification of co-precipitated 2B3 was initially made from the existing database. Only when sequence data from 2B3 was added was a positive match obtained, indicating this sequence differed from group I CbpF and was not in the database (Figure S8). Hence, group II appears to represent a hitherto unrecognised subdivision of autotransporters within *Fusobacterium*.

### Recombinant CbpF–CEACAM1 interactions

Recombinant CbpF was produced from Fn ATCC 25586 and the 2B3 isolate, spanning amino acids from the passenger domain only, to represent groups I and II, respectively. The resulting recombinant proteins lack the β-barrel translocator domain but possess all the YadA-like head domains as well as the putative stalk domain.

**Figure 8.**
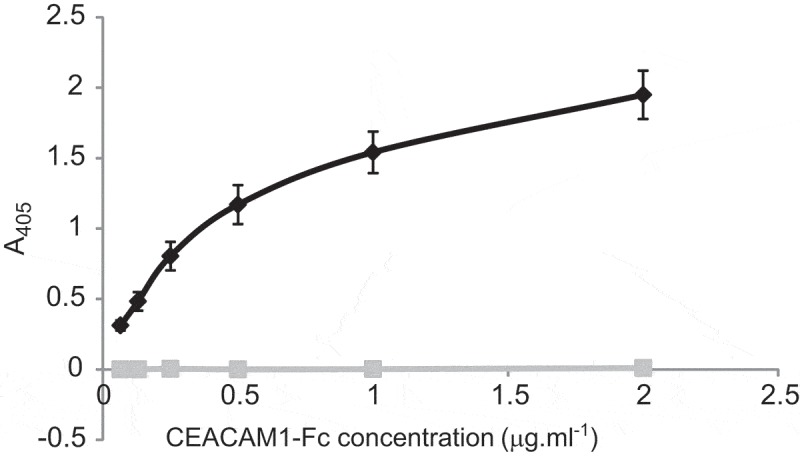
Recombinant CbpF–CEACAM1 interaction. Recombinant CbpF protein was immobilised on ELISA plates and overlaid with CEACAM1-Fc (Black diamonds) or the corresponding I91A mutant (grey squares) Binding was detected using an appropriate alkaline phosphatase-conjugated secondary antibody and chromogenic substrate. Data shown are means of three independent experiments ± SD.

**Figure 9.**
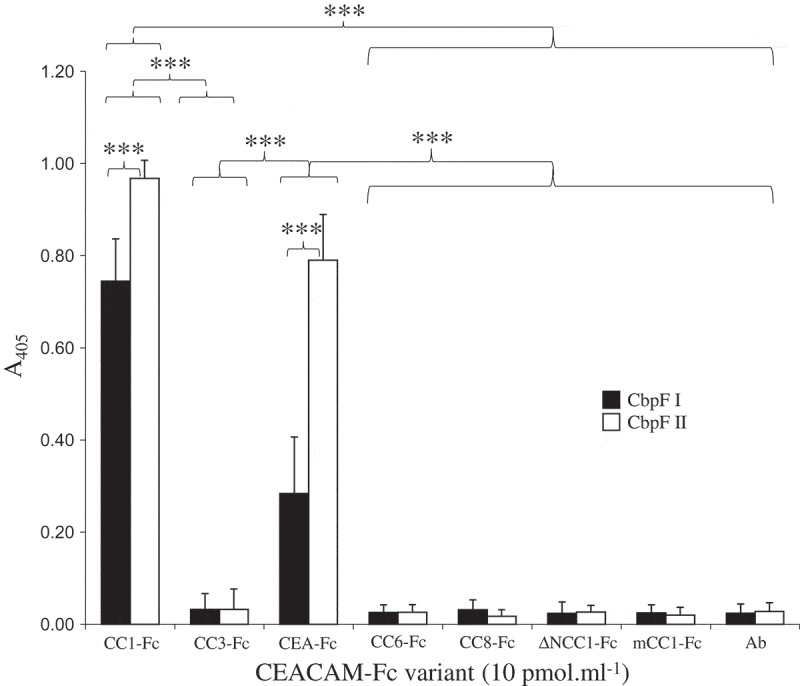
Recombinant CbpF interactions with different CEACAMs. Of purified CbpFI (ATCC25586) and CbpFII (2B3) (3 pmol) immobilised onto ELISA plates. Subsequently, 1 pmol of each CEACAM-F_C_ variant were used followed by an anti-human secondary antibody to detect binding. Each CEACAM-F_C_ conjugate contained all the extracellular IgC- and IgV-like domains except for the ΔN mutant that lacked the N-terminal IgV-like domain. A secondary antibody negative control (*Ab*) was included. Mouse CEACAM1b (mCC1-Fc) and CEACAM8 (CC8-Fc) were also used as these have not previously been shown to bind any human pathogen adhesins. Three independent replicates are shown ± SD. A one-way ANOVA ($F\left(15\right)=370.8$; p<0.0001) followed by a post-hoc Tukey HSD test identified significant differences between CEA and CEACAM1 (***p<0.0001) for CbpF I and II. Both CC1-Fc and CEA bound to CbpF I and II significantly more than all other CEACAM constructs used (p<0.0001).

The protein produced (from 2B3) was able to bind CEACAM1-Fc in a dose-dependent manner up to 2 μg ml^−1^ both by ELISA (Figure 8) and by immunodotblotting (not shown). This recombinant protein was able to bind CEACAM1 in both monomeric and trimeric forms; however, increased binding was apparent to the trimeric form of the protein (Figure S9). Whilst the current study has focussed on CEACAM1, CbpF also has the ability to bind to CEA (Figure S10), albeit to a varying degree with the group I protein displaying significantly less binding compared to group II CbpF, about 50% as well. Moreover, the CbpF from group II displayed significantly higher binding to CEACAM1-Fc than group I CbpF, though the difference was not as great, about 75%. Neither class of CbpF showed the ability to bind other CEACAM constructs including CEACAM3-Fc, CEACAM6-Fc, CEACAM8-Fc, or murine CEACAM1b (mCEACAM1b-Fc; Figure 9).

**Figure 10.**
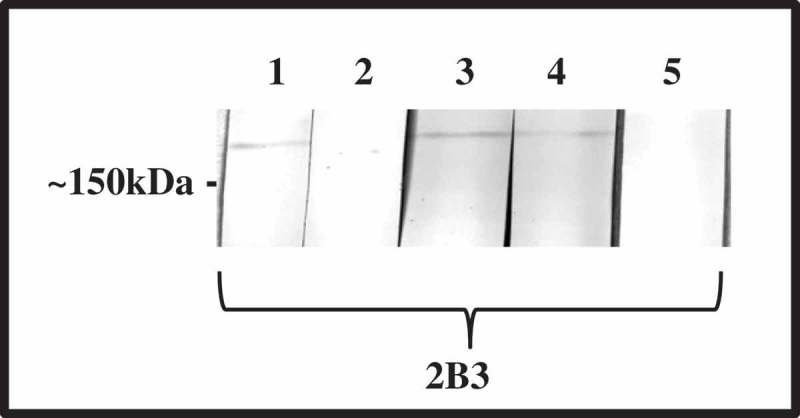
Inhibition of N-Fc binding to a clinical isolate of Fn. Lysate 2B3 was separated by SDS-PAGE using 5% trench well gels. Following Western blotting, strips were but and overlaid with CEACAM1 N-Fc alone (0.2 μg ml^−1^; lane 1), in the presence of YTH71.3 (anti-CEACAM N-domain antibody raised in rat, 10 μg ml^−1^; lane 2), in the presence of control antibody Kat4c (anti-CEACAM antibody raised in mouse not recognising N-Fc, 10 μg ml^−1^; lane 3). Lane 4 strip was overlaid with CEACAM1 N-Fc (0.2 μg ml^−1^; lane 1) in the presence of a rat isotype control antibody. Lane 5 strip was overlaid with CD33-Fc (0.2 μg ml^−1^) as a negative control. Receptor binding was detected using anti-human-Fc conjugated to alkaline phosphatase and developed using NBT/BCIP. N-Fc binding to both clinical isolates was completely inhibited in the presence of YTH71.3 but not the control antibody Kat4c or the rat isotype control. Image is representative of two independent experiments.

**Figure 11.**
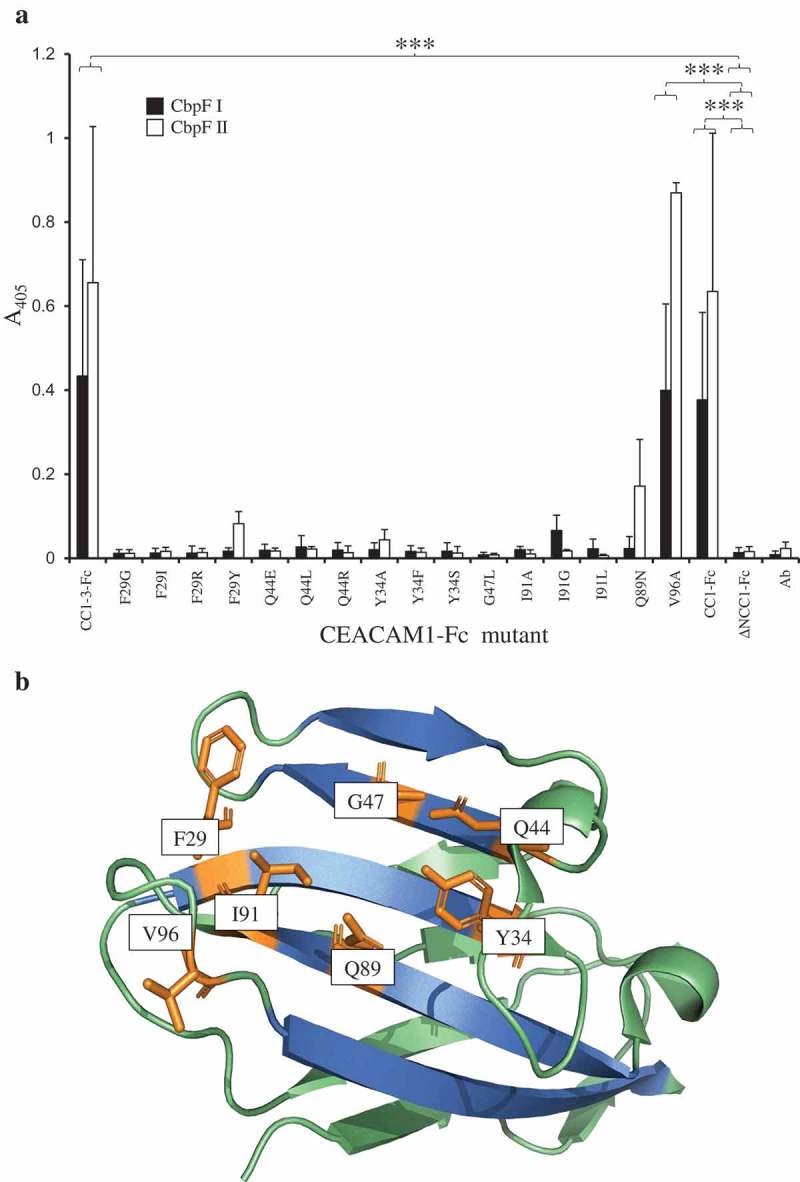
Recombinant CbpF interactions with CEACAM1 mutant library. (a) Equimolar amounts of each CbpF protein (3 pmol) were overlaid with 15 fmol of CEACAM1-3-Fc (CC1-3-Fc) mutants in ELISA assays. In addition, CEACAM1-4-Fc (CC1-Fc) and CEACAM1-A1BA2-Fc (ΔNCC1-Fc) were used as positive and negative controls, respectively. In addition, an anti-human IgG-Fc-AP only control was used (*Ab*). The ELISA was developed for 5 hrs at 37°C using the SigmaFast® kit. (a) two-way ANOVA followed by a post-hoc TukeyHSD test was used to identify significant difference between conditions (N-terminal mutant: $F\left(19\right)=42.86$, p<0.0001. Three independent replicates are shown ± SD. (b) Structure of CEACAM1 N-domain displaying relative positions of the amino acids (shown in orange) which were mutated and used for this study. The CFG face is shown in blue, whilst the remainder of the structure is shown in green.

In keeping with other bacterial pathogens studied to date which bind to CEACAM1 [5,6,10], binding could be inhibited by the N-domain binding antibody YTH71.3 (Figure 10). Examination of CEACAM1-Fc IgV-like domain mutants confirmed that residues on the CFG face of CEACAM1 were critical for adhesion. Mutating the residues F29 or Q44 to amino acids found naturally in other CEACAMs consistently reduced adhesion to background (Figure 11(a)). For reference, the relative positions of the mutated amino acids are indicated in Figure 11(b).

These data indicate that the passenger domain of CbpF contains the CEACAM-binding motif as might be expected and appears to share an overlapping binding region on the N-domain of CEACAM1 with other pathogenic bacterial CEACAM-binding ligands. Functional recombinant CbpF will facilitate future studies on the precise nature of CEACAM–CbpF interactions.

## Discussion

Here we report that *Fusobacterium* binds to human CEACAM1 via a trimeric autotransporter which we have named CbpF. This protein exists as a ~50 kDa subunit but runs as a stable trimer during electrophoresis and Western blotting. Other reported adhesins for Fn include RadD, a putative autotransporter protein implicated in arginine-dependent interspecies interactions and biofilm formation of Fn [42]. A small α-helical protein FadA has been demonstrated to target human vascular endothelial cadherin and subsequently enables endothelial tight junction penetration by Fn and other species [43]. Fn species also express a porin, FomA which has been shown to bind to statherin [44] and also the human immunoglobulin Fc region [45]. Given our CEACAM1 construct was conjugated to human IgG-Fc, there was potential for IgG- Fc-FomA binding to occur. Initial studies identified CEACAM1-Fc binding in Fv. No *fomA* gene appears to be present in the genome for ATCC 49256, the strain of Fv used in this study, thus expression of FomA may vary between strains. Using equivalent amounts of CEACAM1-Fc and CD33-Fc, we always observed CEACAM1-Fc binding to be higher than that of the control protein in immunodot blots, Western blots and ELISA-based assays employed in this study. In addition, commercially available CEA (which lacks Fc) bound to CbpF (Figure S10) and the CEACAM N-domain specific antibody YTH71.3 blocked the CbpF–CEACAM1-Fc interaction. CbpF therefore appears to specifically target CEACAMs as do other TAAs such as UspA1 and UspA2V expressed by *M. catarrhalis* [5,14].

Little sequence identity was found to exist between CbpF and the other known CEACAM-binding ligands of diverse bacterial species including the type-1 fimbriae of *E. coli* and *Salmonella typhimurium*, shown to bind to oligosaccharides on CEACAMs [46] or the Afa/Dr family proteins of *E. coli* [47]. Inhibition of CbpF–CEACAM1-Fc binding by YTH71.3 suggest Fn targets the same region of the non-glycosylated face of CEACAM1 targeted by Opa, P5, and UspA proteins [10,35,36].

The diverse adhesive functions of the trimeric autotransporter family are conveyed by the passenger domain of these proteins [reviewed in 19]. We produced recombinant CbpF passenger domain from the clinical strain 2B3 and Fn ATCC 25586 lacking the β-barrel (which can lead to solubility issues) and lacking the signal peptide (to prevent cleavage of the N-terminal His tag needed for purification). Recombinant CbpF migrated predominantly in monomeric form following heating, unlike native CbpF expressed by fusobacterial species. The decreased tendency to oligomerise could be attributed to the truncated form of the recombinant molecule lacking the β-barrel region of the protein. CEACAM binding to the monomeric form of CbpF was evident in Western blot overlays. Heat alone is sufficient to convert some TAAs to monomeric form such as UspA1 of *M. catarrhalis* [48]. Others including UspA2 of *M. catarrhalis* have been denatured using formic acid [32]. Unlike the CbpF monomer produced using formic acid, monomeric recombinant CbpF was able to bind to CEACAM1-Fc. This suggests formic acid exposure resulted in the loss of a conformational epitope still present within the monomeric recombinant even after SDS-PAGE and Western blotting. This is in contrast to the observations made with the CEACAM1 binding TAAs of *M. catarrhalis*, UspA1 and UspA2V, which retain the ability to bind to CEACAM even following treatment with formic acid [14]. Overall CbpF–CEACAM interaction appears, therefore, to be more dependent on conformation than the CEACAM-binding UspA proteins of *M. catarrhalis*. We confirm that in keeping with other TAAs, the adhesive function of CbpF resides in the passenger domain.

Sequencing of CbpF from a number of *Fusobacterium* clinical isolates revealed two distinct groups (I and II) which differed mainly in the number of head repeat regions. Assays to detect binding to other CEACAMs (CEACAM1, 3, 5, 6, 8, and murine CEACAM1) only showed demonstrable binding for both group I and II CbpFs to CEACAM and CEA. While group II CbpF was bound CEA and CEACAM1 to the same level, group I CbpF favoured CEACAM1 showing significantly reduced binding compared to CEA (Figure 9).

The phenylalanine at position 29 and glutamine at position 44 on the CFG face of CEACAM1 are the only two unique residues shared between CEACAM1 and CEA, F29 is also found in CEACAM3, whilst Q44 is not found in any other CEA-family member. Altering the F29 and Q44 residues to naturally occurring alternatives found within other CEACAMs completely disrupts binding (Figure 11). Additionally, mutants from other areas on the CFG face were shown to negate adhesion, such as Y34, G47, Q89, and I91. Interestingly, Q89N displayed reduced, but higher than background, levels of adhesion to group II CbpF and no binding at all to group I. The higher binding of V96A mutant to CbpFII compared to CbpFI could implicate V96 in the CbpF binding surface on CEACAM1. However, no significant difference was observed between V96A and CC1-Fc in binding to recombinant CbpFI or CbpFII. As the IgV-like N-terminal domain of all CEACAMs is highly conserved, and that CbpFs have only been seen to bind CEACAM1 and CEA, this suggests that these proteins have specifically evolved to target these proteins while avoiding other highly similar receptors such as CEACAM3. This is contrary to UspA1 for example, which has been shown to bind to CEACAM3 and -6 as well as CEACAM1 and -5 [49,50]. This could allow CbpF to avoid activating neutrophils where CEACAM3 is exclusively located, where CEACAM3 induction can lead to a proinflammatory effect via its immunoreceptor tyrosine-based activation motif (ITAM)-like domain [49,51].

The lack of a conserved CEACAM-binding motif between bacterial species render an *in silico* identification of the CEACAM-binding domain of CbpF redundant. Based on previous experience of CEACAM-binding ligands, it is not always the most conserved region of the protein within each species which is responsible for CEACAM binding. For example, the more diverse regions of Opa residing within two surface-exposed loops mediate neisserial interaction to CEACAMs [52]. Conversely, in the case of UspA1 proteins, a critical ~20 amino acid region within the coiled-coil stalk mediates CEACAM1 binding [36]. Within the published genome of Fn ATCC 25586, two other putative TAAs were identified, FN0471 and FN0735. Differences in the sequences of FN0471 and FN1321 may be useful in aiding the elucidation of the CEACAM-binding region of CbpF. However, if the corresponding proteins are not expressed, sequence comparison is meaningless. Within our strain collection, two strains (2B24 and 2B28) produced a PCR product with CbpF primers (although slightly larger than the products for type-I and type-II CbpF) yet did not bind CEACAM1. Besides differences in sequence, one potential explanation for strains 2B24 and 2B28 proteins lacking CEACAM binding is phase variation.

In CbpF, we observed antigenic variation with a number of insertion/deletion events comparing the size of the head between group I and group II CbpF as well as individual amino acid changes. *F. nucleatum* has a very low GC content of ~27% [53] containing numerous polymeric nucleotide tracts which could lead to phase variation of genes. Whilst potential phase variation of other autotransporters of *F. nucleatum* has been described [41], to date no studies have demonstrated phase variation in *F. nucleatum* TAAs. Therefore, future studies will compare the sequences of CbpF and related homologues with their expression levels and their ability to bind to CEACAM1.

*F. nucleatum* appears to have a uniquely adaptable role in interactions with both the host and other microbes. It is an important component of dental plaque due to a promiscuous ability to coaggregate with many different species of oral bacteria [54]. Fn is often portrayed in the literature as a secondary coloniser and an important link in biofilm establishment [55]. Fn is a commonly isolated periodontal pathogen from clinical infections at other body sites including bacteraemia, amniotic fluid as well as intestinal tissue associated with inflammatory bowel disease [56–58]. Such variety in isolation site suggests that this species has the capacity to interact directly with diverse human cells and tissues.

Of interest to these studies is the fact that other bacterial species able to utilise CEACAMs for adherence also use them for cellular invasion and tissue penetration; this is the case with *N. meningitidis* [59]. In addition, CEACAM engagement by bacterial pathogens leads to reduced epithelial shedding [60]. Notably, *Helicobacter pylori* has recently been shown to engage human CEACAM1 via the surface protein HopQ [33,61,62]. Engagement of HopQ facilitates the translocation of the virulence factor CagA into host cells promoting a proinflammatory response [62].

The identification of the CEACAM1-binding TAA expressed by a subset of *Fusobacterium* isolates will enable the role of CbpF in disease to be studied in more detail. Such studies may be of particular importance to cancer as fusobacterial species have been associated with colorectal cancer and resistance to therapies [63,64]. *Fusobacterium* has also been associated with oral carcinomas [64]. Through better understanding of the defined interactions in such disease processes, future studies may inform potential therapeutic strategies to reduce the global burden of disease caused by *Fusobacterium* spp. but also those other pathogens that require interaction with this organism to aid colonisation and subsequent pathology.
