# Supplementary material for: Fusobacterium spp. target human CEACAM1 via the trimeric autotransporter adhesin CbpF
Source: J Oral Microbiol. 2019 Jan 24;11(1):1565043. doi: 10.1080/20002297.2018.1565043 (PMC6346709; doi:10.1080/20002297.2018.1565043)
Supplement: Supplemental Material [file ZJOM_A_1565043_SM8386.pdf]

Table S1. Oral bacterial isolates used in CEACAM binding screen.

SPECIES	DESIGNATION
Aggregatibacter actinomycetemcomitans	Y4
Aggregatibacter actinomycetemcomitans	P34 (B3036)
Aggregatibacter actinomycetemcomitans	P39 (B3071)
Aggregatibacter actinomycetemcomitans	P44 (B3104)
Actinomyces gerencseriae	ATCC 23860
Actinomyces naeslundii	NCTC 10301
Actinomyces odontolyticus	NCTC 9935
Actinomyces viscosus	NCTC 10951
Staphylococcus cohnii	ATCC 49331
Bacteroides fragilis	ATCC 25285
Campylobacter gracilis	ATCC 33236
Dialister pneumosintes	ATCC 33048
Campylobacter ureolyticus	NCTC 10939
Campylobacter concisus	NCTC 11485
Campylobacter rectus	NCTC 11489
Campylobacter rectus	ATCC 33238
Capnocytophaga gingivalis	ATCC 33624
Capnocytophaga ochracea	ATCC 27872
Capnocytophaga sputigena	ATCC 33612
Eikenella corrodens	NCTC 10596
Eggerthella lenta	NCTC 11813
Eubacterium nodatum	ATCC 33099
Mogibacterium timidum	ATCC 33093
Fusobacterium nucleatum	ATCC 49256
Fusobacterium periodonticum	ATCC 33693
Parvimonas micra	ATCC 33270
Peptostreptococcus anaerobius	NCTC 11460
Porphyromonas gingivalis	ATCC 33277
Prevotella intermedia	ATCC 25611
Prevotella nigrescens	ATCC 25261
Prevotella buccalis	ATCC 35310
Prevotellacorporis	ATCC 33547
Prevotella denticola	ATCC 35308
Prevotella loescheii	NCTC 11321
Prevotella melaninogenica	ATCC 25845
Porphyromonas asaccharolytica	ATCC 25260
Prevotella oris	ATCC 33593
Prevotella oulorum	ATCC 43324
Alloprevotella tannerae	ATCC 51259
Streptococcus cristatus	CC5A
Streptococcus gordonii	ATCC 10558
Streptococcus gordonii	DL1
Streptococcus gordonii	Wild type DL1
Streptococcus gordonii	Mutant DL1
Streptococcus intermedius	ATCC 27335
Streptococcus anginosus	NCTC 10713
Streptococcus milleri	NCTC 10708
Streptococcus mitis	NCTC 10712
Streptococcus mitis	NCTC 12261
Streptococcus mutans	NCTC 10449 D
Streptococcus mutans	UAB 159 type strain
Streptococcus oralis	NCTC 11427
Streptococcus salivarius	NCTC 8606
Streptococcus sanguinis	NCTC 7863
Streptococcus sanguinis	NCTC 10904
Streptococcus sobrinus	6715
Veillonella atypica	NCTC 11830
Veillonella dispar	NCTC 11831
Veillonella alkalescens	NCTC 11809
Campylobacter curvus	ATCC 35224
Wolinella Succinogenes	NCTC 11488

Figure S1.

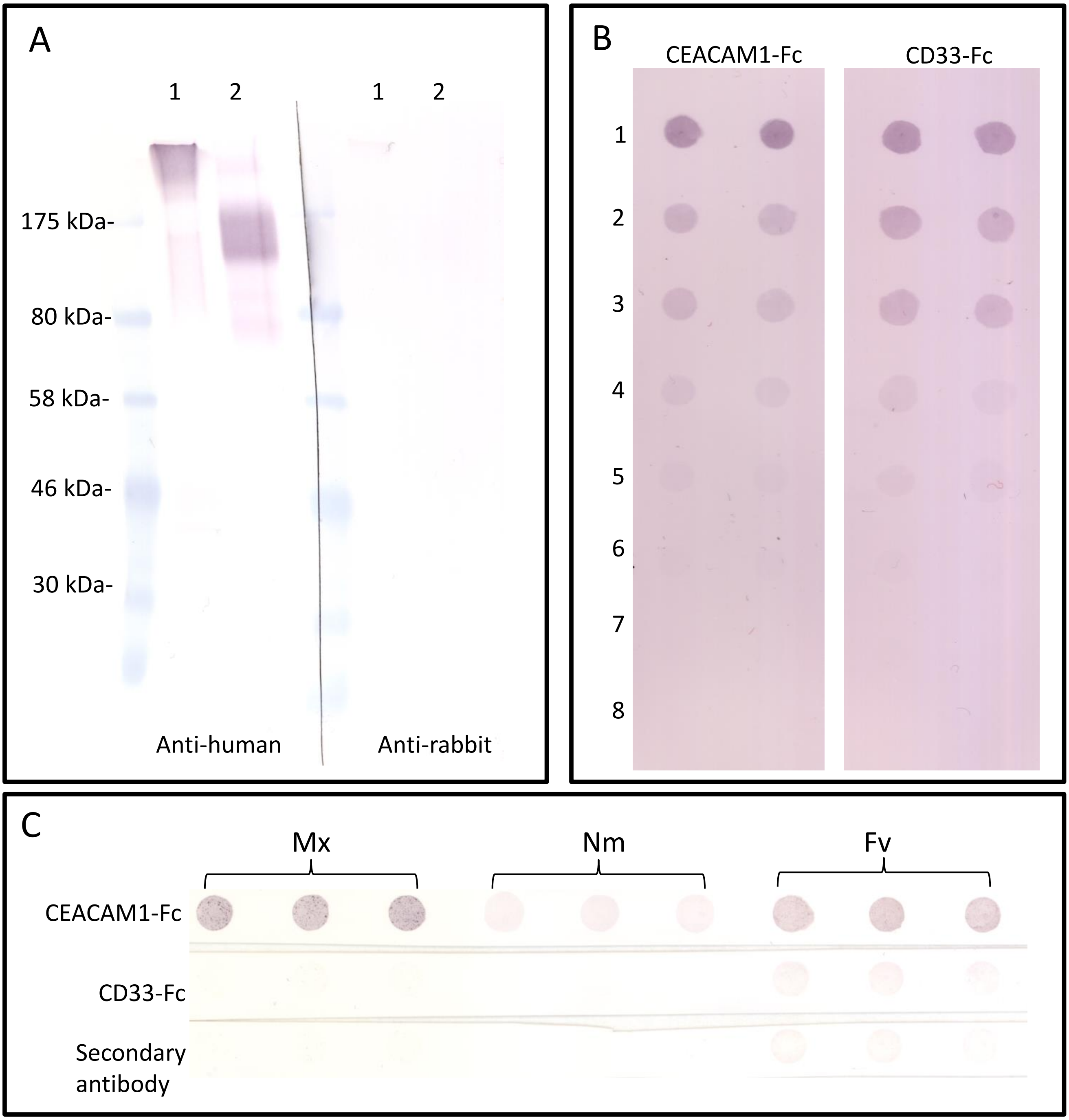

Figure S1. A) Comparison of CEACAM1-Fc and CD33-Fc dimers by anti-human-Fc antibody. 0.5 µg of CEACAM1-Fc (lane 1) or CD33-Fc (lane 2) were separated by SDS-PAGE and Western blotted. Blots were overlaid with anti-human-Fc alkaline phosphatase conjugated antibody or anti rabbit Ig isotype control. Bands of similar intensity and appropriate size for the both CEACAM1-Fc and CD33-Fc dimers were detected by anti-human-Fc, but not the anti-rabbit control antibody. B) Dotimmunoblot of CEACAM1-Fc and CD33-Fc. CEACAM1-Fc and CD33-Fc were double diluted from a stock solution of 1µg.ml^{-1} and 30 µl of the dilution series dotted in duplicate under vacuum onto nitrocellulose membrane (1-8). The membrane was overlaid with anti-human-Fc conjugated to alkaline phosphatase. C) Representative immunodotblot of CEACAM1-Fc binding to oral bacterial species. Bacterial isolates standardised by spectrophotometery, applied to nitrocellulose, and overlaid with CEACAM1-Fc (1µg.ml^{-1}). Mx: CEACAM-binding UspA1 expressing *M. catarrhalis* strain MX1 (positive control), Nm: Opa negative variant *N. meningitidis* strain C751 (negative control), Fv: *Fusobacterium vincentii*.

Figure S2

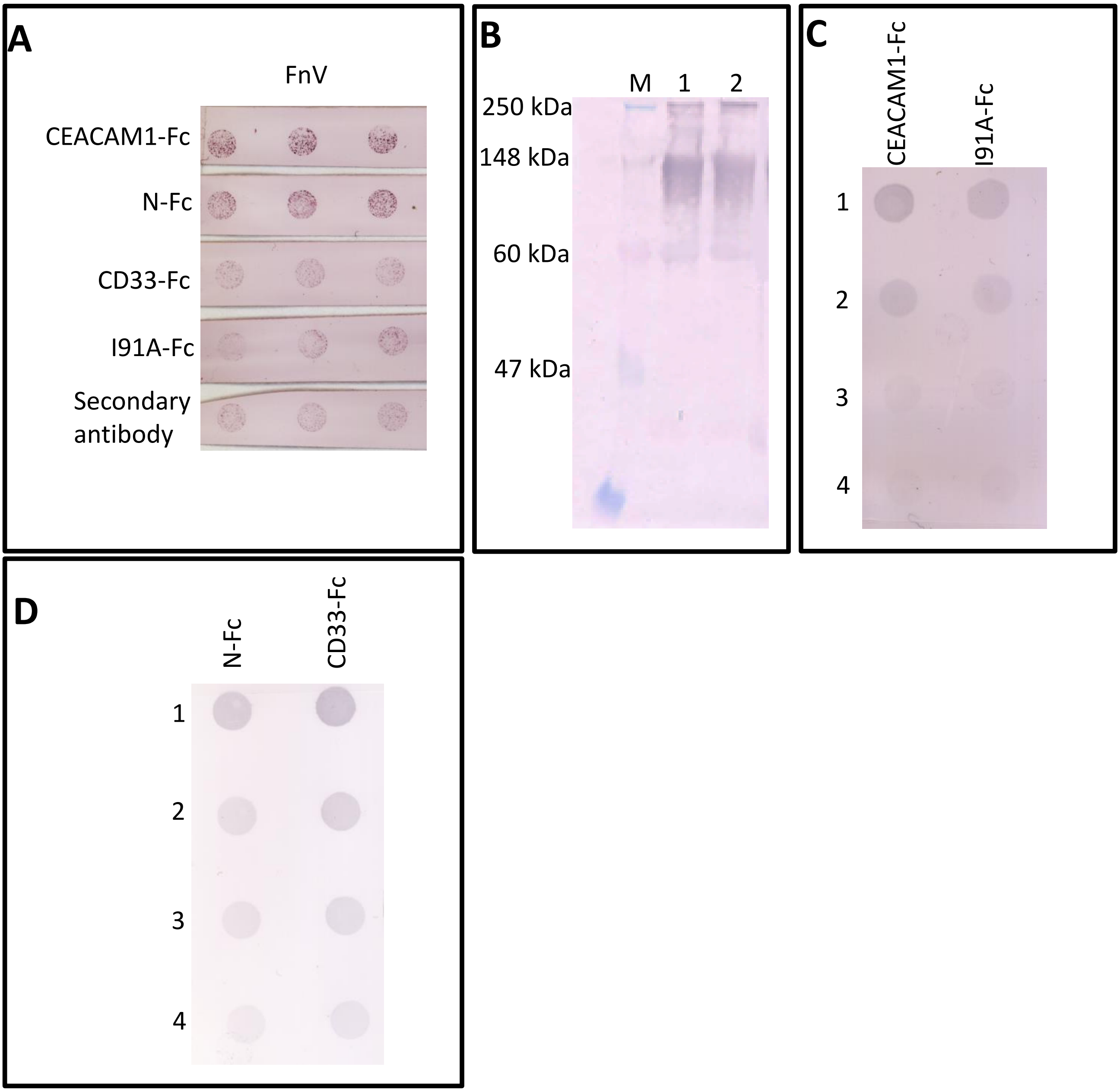

Figure S2. CEACAM1 N-domain binding to *F. vincentii*. A) Representative immunodotblot of Fv overlaid with CEACAM1 (CC1-Fc), CEACAM1 N-domain (N-Fc), CEACAM1 I91A construct (I91A-Fc) or CD33 construct (CD33-Fc) used as a negative control. B) Western blot of CEACAM1-Fc (lane 1) and I91A-Fc (lane2; both 0.5µg.ml^{-1}) showing equivalent amounts of each monomeric form detected by anti-human-Fc alkaline phosphatase conjugate. C) Immunodotblot of –Fc construct. CEACAM1-Fc and I91A-Fc were double diluted from a stock solution of 1µg.ml^{-1} and thirty µl of the dilution series dotted in triplicate under vacuum onto nitrocellulose membrane (1-4). D) Immunodotblot –Fc constructs. N-Fc and CD33-Fc were double diluted from a stock solution of 1µg.ml^{-1} and 30 µl of the dilution series dotted under vacuum onto nitrocellulose membrane (1-4). The membrane was overlaid with anti-human-Fc conjugated to alkaline phosphatase. In the case of panels B-D) note the equivalent detection in each comparison by the anti-human-Fc alkaline phosphatase conjugated antibody.

Figure S3.

Signal P predicted signal peptide

```
1   MKKFVSLKLIVFSFILVAGSVSYSAAPVIKAGTATDSTEAGVDNVANGVK FN1499
1   MKKFVSLKLIVFSFILVASSVSFS-VPVIQGGTGSDSTVAGIGNDASGEN FNV1729

51  SSAFGYDNKAIEKESSAFGTGNRATGEFSSAFGFHNIASKIHSSAFGSNN FN1499
50  SSAFGFHN--T------------ASGKFSSAFGFRNIASKLRSSAFGTGN FNV1729

101 AADGVNSSAFGFKNTVSGFNSSAFGSQYQVTGNFSGAFGMGEFNGQYQYK FN1499
86  KADGEDSSAFGSLNIASGKFSSAFGSQYEVTGNSSGAFGVGEFNGSYQYK FNV1729

151 NEGNNSYMIGNKNKIASGSDDNFILGNNVHIGGGINNSVALGNNSTVSAS FN1499
136 NEGNNSYMIGNKNKIASGSNDNFILGNNVHIGGGINNSVALGNNSTVSAS FNV1729

201 NTVSVGSSTLKRKIVNVGDGAISANSSDAVTGRQLYSGNGIDTAAWQNKL FN1499
186 NTVSVGSSTLKRKIVNVGDGEISASSTDAVTGRQLYSGNGIDTAAWQNKL FNV1729

251 NVTRKNDYKDANDIDVNKWKAKLGVGSGGGGGAPVDAYTKSEADNKFANK FN1499
236 NVTKKNDYKDANDIDVNKWRTKLGVGSGGGGGAPVDSYTKSEADNKFANK FNV1729

301 TDLNDYTKKDDYKDANGIDVDKWKAKLGTGAGTADIENLRNEVNEKIDDV FN1499
286 TDLDNYTKKDDYKDANGIDVDKWKAKLGTGAGTADIENLRNEVNEKIDDV FNV1729

351 KDEVRTVGSLSAALAGLHPMQYDPKAPVQVMAALGHYRDKQSVAVGASYY FN1499
336 KDEVRTVGSLSAALAGLHPMQYDPKAPVQVMAALGHYRDKQSVAVGASYY FNV1729

401 FNDRFMMSTGIALSGEKRTKTMANVGFTLKLGKGSGVTYDETPQYVVQNE FN1499
386 FNDRFMMSTGIALSGEKRTKTMANVGFTLKLGKGSGVTYNETPLYTIQDE FNV1729

451 VKRLTVEN-------QELKERVRNLEEKLNMLLKNK               FN1499
436 VKRLTVENNKQAKENQELKERVRNLEEKLNMLLKNK               FNV1729
```

Figure S3. Alignment of CbpF from Fn and Fv identified following N-terminal sequencing of the co-immunoprecipitated CEACAM1-binding ligand. Proteins sequences retrieved from the NCBI database were aligned pairwise using the Clustal W alignment method using MegAlign from DNASTAR. The overall sequence identity is 88.8% between the two proteins. The location of the 15-mer peptide used to raise antiserum KW15 is highlighted in yellow.

Figure S4.

Percent Identity

Divergence		1	2	3	4	5		
	1		88.8	60.2	57.6	60.9	1	FN1499.pro
	2	12.2		59.0	56.2	59.0	2	FNV1729.pro
	3	56.1	58.6		77.6	93.0	3	FNP1391.pro
	4	61.6	64.7	26.6		78.8	4	FN0471.pro
	5	54.8	58.6	7.3	24.9		5	FN0735.pro
		1	2	3	4	5		

Figure S4. Relatedness of CbpF type autotransporters in species of Fusobacterium within the GenBank database. Proteins sequences were aligned pairwise using the Clustal W alignment method using MegAlign from DNASTAR. Percent identity of each sequence pair (as indicated) is shown in the top right hand section and the divergence of each sequence pair is shown in the bottom left hand section of each table. Percent identity compares sequences directly whereas divergence is calculated from sequence pairs based on their reconstructed phylogeny.

Figure S5

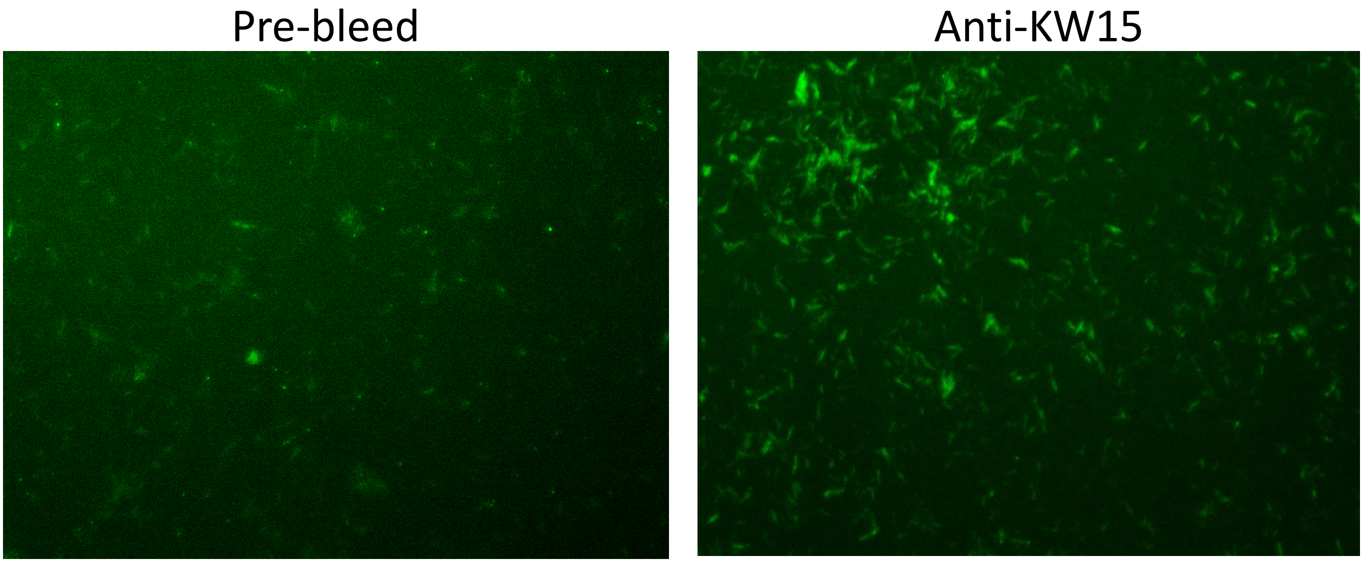

Figure S5. Fastidious anaerobe agar cultured Fv were immobilised onto poly-L-lysine coated tissue culture plates for 30 min at room temperature. Bacteria were overlaid with either anti-KW15 or control pre-bleed serum as indicated and washed to remove unbound antibody. Anti-KW15 was detected using AlexaFluor-488 conjugated anti-rabbit antibody (Molecular probes Inc.) and unbound antibody removed by washing. Bacteria were subsequently fixed in paraformaldehyde and examined using a Olympus IX70 microscope with fluorescent attachment. Note the detection of surface exposed CbpF by anti-KW15 antiserum above pre-bleed control levels.

Figure S6.

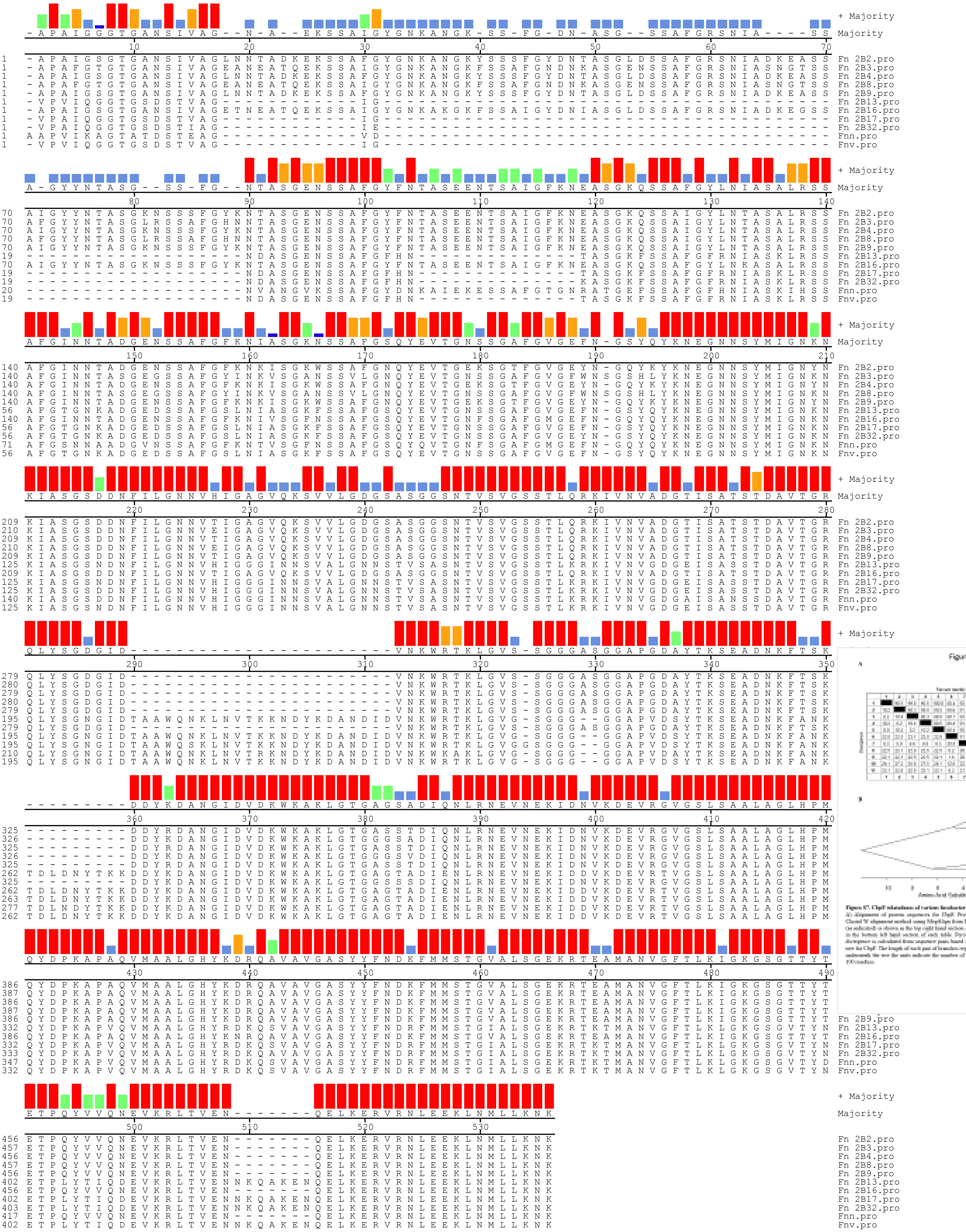

Figure S6. Alignment of CbpF from clinical Fusobacterium isolates compared to Fn and Fv. Proteins sequences retrieved from the NCBI database were aligned pairwise using the ClustalW alignment method using MegAlign from DNASTAR. The top line below the histogram boxes indicates the consensus sequence (majority). The numbering above the alignment indicates positions within the consensus sequence and the numbers to the left hand side refer to the individual sequences. The overall consensus strength is shown by coloured histograms above each aligned amino acid; increasing height and colour indicates increased consensus strength (blue<green<orange<red).

Figure S7.

A

Percent Identity (upper right); Divergence (lower left)

	1	2	3	4	5	6	7	8	9	10	11		
**1**		90.5	99.8	90.5	100.0	80.4	93.9	80.6	80.9	78.0	80.1	**1**	2B2
**2**	10.2		90.3	99.8	90.5	80.6	91.7	80.9	80.9	77.3	80.4	**2**	2B3
**3**	0.2	10.4		90.3	99.8	80.1	93.7	80.4	80.6	78.2	80.4	**3**	2B4
**4**	10.2	0.2	10.4		90.5	80.4	91.7	80.6	80.6	77.0	80.1	**4**	2B8
**5**	0.0	10.2	0.2	10.2		80.4	93.9	80.6	80.9	78.0	80.1	**5**	2B9
**6**	22.8	22.5	23.1	22.8	22.8		81.9	99.8	98.2	88.4	99.8	**6**	2B13
**7**	6.3	8.8	6.6	8.8	6.3	20.8		82.1	81.9	80.6	81.6	**7**	2B16
**8**	22.5	22.1	22.8	22.5	22.5	0.2	20.5		98.4	88.2	99.6	**8**	2B17
**9**	22.1	22.1	22.5	22.5	22.1	1.8	20.8	1.6		87.7	98.0	**9**	2B32
**10**	26.1	27.2	25.8	27.5	26.1	12.6	22.5	12.9	13.4		88.6	**10**	Fn
**11**	23.1	22.8	22.8	23.1	23.1	0.2	21.1	0.4	2.0	12.4		**11**	Fv
	**1**	**2**	**3**	**4**	**5**	**6**	**7**	**8**	**9**	**10**	**11**		

B

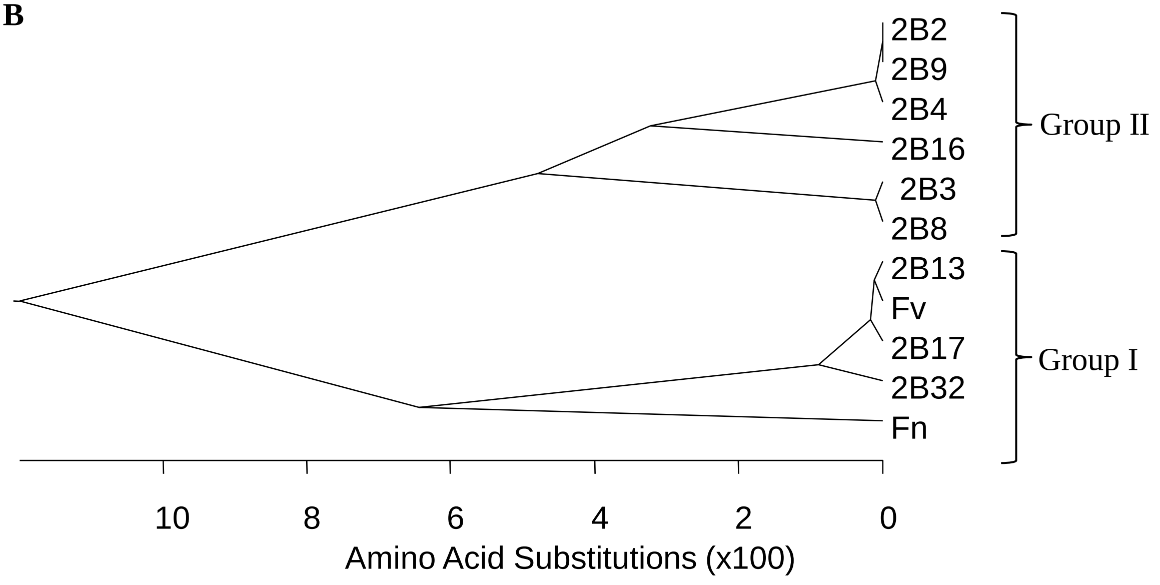

Figure S7. CbpF relatedness of various fusobacterial isolates

A) Alignment of protein sequences for CbpF. Proteins sequences were aligned pairwise using the Clustal W alignment method using MegAlign from DNASTAR. Percent identity of each sequence pair (as indicated) is shown in the top right hand section and the divergence of each sequence pair is shown in the bottom left hand section of each table. Percent identity compares sequences directly whereas divergence is calculated from sequence pairs based on their reconstructed phylogeny. B) Phylogenetic tree for CbpF. The length of each pair of branches represents the distance between sequence pairs, while underneath the tree the units indicate the number of substitution events as amino acid substitutions per 100 residues.

Figure S8

A

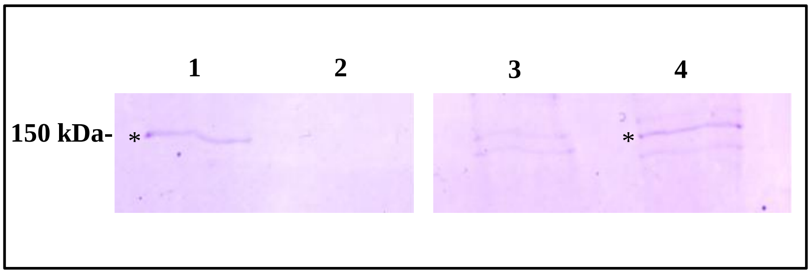

B

Accession	Coverage	# PSMs	# Peptides	# AAs	MW [kDa]	calc. pI	Score	Description
Q8RIS0	10.65	22	6	479	50.8	8.88	93.83	Cell surface protein OS=Fusobacterium nucleatum subsp. nucleatum (strain ATCC 25586)
Q8RIP5	1.68	4	3	2143	221.5	9.13	23.16	Fusobacterium outer membrane protein family OS=Fusobacterium nucleatum subsp. nucleatum (strain ATCC 25586)
Q8RGZ2	9.33	1	1	150	17.2	8.94	10.51	Putative uncharacterized protein OS=Fusobacterium nucleatum subsp. nucleatum (strain ATCC 25586)

C

Accession	Coverage	# PSMs	# Peptides	# AAs	MW [kDa]	calc. pI	Score	Description
2B3 CbpF	45.75	95	17	518	53.6	8.78	383.08	2B3 CbpF
Q8RIS0	10.65	22	6	479	50.8	8.88	93.83	Cell surface protein OS=Fusobacterium nucleatum subsp. nucleatum (strain ATCC 25586)
Q8RIP5	2.10	5	4	2143	221.5	9.13	23.16	Fusobacterium outer membrane protein family OS=Fusobacterium nucleatum subsp. nucleatum (strain ATCC 25586)

Figure S8. Validation of group II CbpF sequence.
A) Coomassie stained Western blot of CbpF from 2B3 co-precipitated with N-Fc. A Protein band of ~150kDa was present (*) when co-precipitated with N-Fc (lane 1) but not with protein A-sepharose control (lane 2). The same molecular weight band was observed in the post protein A-sepharose column lysate (* lane 4) but appeared depleted in the material post N-Fc protein A-sepharose column (lane 3). Protein from a corresponding SDS-PAGE gel was analysed by LC MS/MS as a service by the University of Bristol Proteomic Facility. Briefly, Gels were subjected to in-gel trypsin digestion using an automated digestion unit (Digilab, UK). Peptides were fractionated using a Dionex Ultimate 3000 nano HPLC system and ionised by nano-electrospray ionisation at 2.3kV. Tandem mass spectrometry was carried out using an LTQ-Orbitrp Velos mass spectrometer (Thermo Scientific). The top twenty ions in each cycle were selected for MS/MS in the LTQ linear ion trap. Data were acquired using Xcaliber software. Searches were performed against the UniProt *Fusobacterium nucleatum* subspecies nucleatum database alone (B) or with the addition of 2B3 CbpF (C) and the top three matches shown in each case. The reverse database search option was enabled and all peptide data was filtered to satisfy false discovery rate (FDR) of 5%. The Proteome Discoverer software generates a reverse "decoy" database from the same protein database and any peptides passing the initial filtering parameters that were derived from this decoy database are defined as false positive identifications. The minimum cross-correlation factor (Xcorr) filter was readjusted for each individual charge state separately to optimally meet the predetermined target FDR of 5% based on the number of random false positive matches from the reverse decoy database. Thus each data set has its own passing parameters. The increased sequence coverage, peptide spectral matches (PSMs) and overall score was greatly improved (~4X) when the derived 2B3 CbpF sequence was added to the searched database relative to the group I CbpF from Fnn. The improved technology of the LTQ-orbitrap coupled with sequence differences from the database may account for the lack of CbpF identification initially using MALDI-TOF based tryptic peptide fingerprinting.

Figure S9.

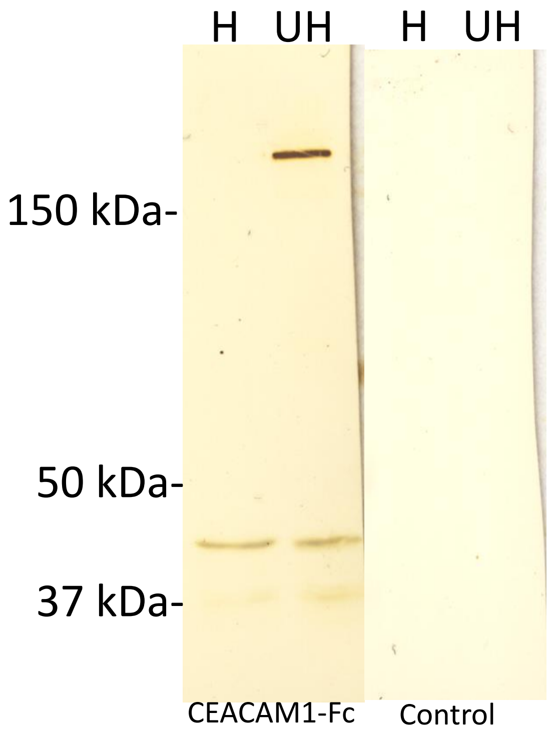

Figure S9. Binding of recombinant CbpF (rCbpF) to CEACAM1-Fc. 1µg of recombinant protein was either not heated (UH), or heated (H) at 100°C for 5min. Following overlay with CEACAM1-Fc binding was observed to monomeric rCbpF but also trimeric CbpF. It is noteworthy that the trimeric CbpF appears to have a higher avidity for CEACAM1-Fc than the monomeric form which may be indicative of some conformational role depending on the trimer for optimal CEACAM binding to occur.

Figure S10

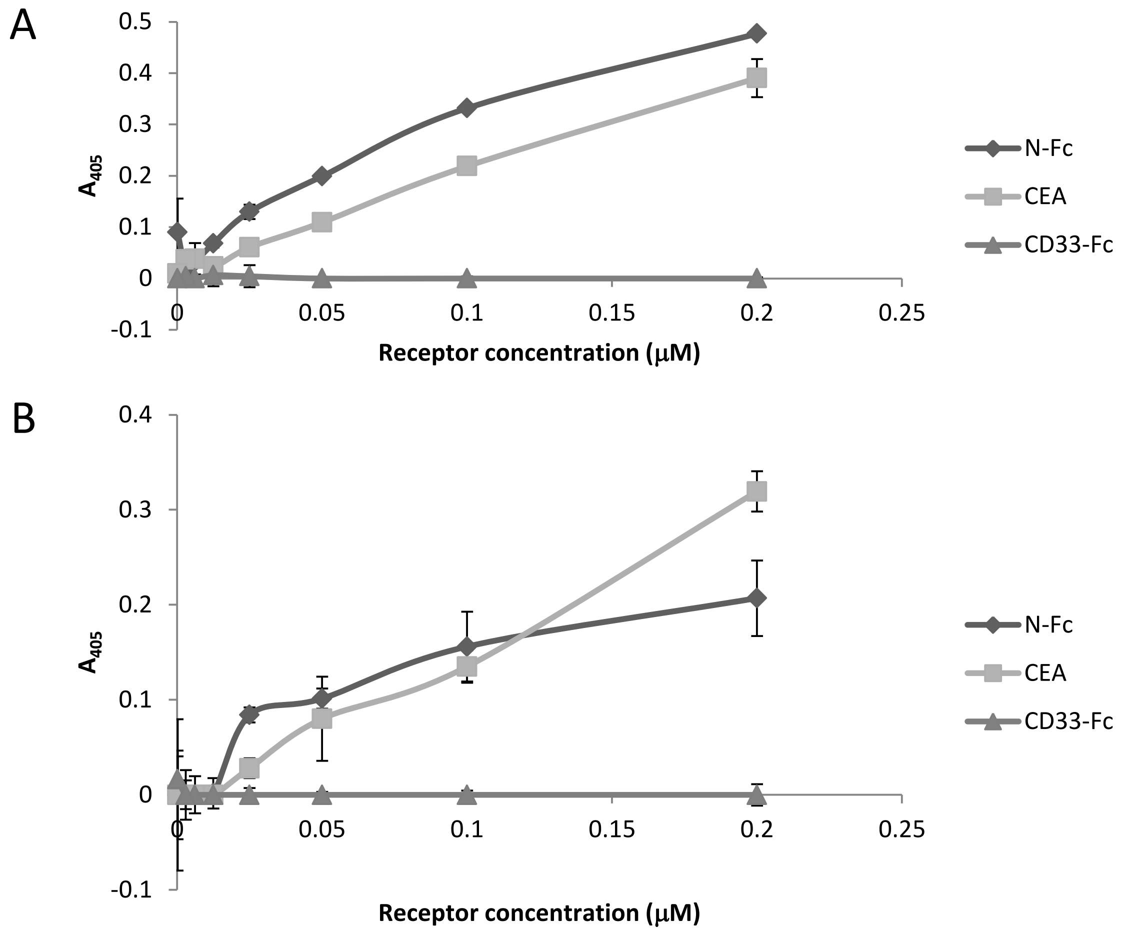

Figure S10. Binding of N-Fc and CEA to CbpF. Elisa plates were coated with either 2mM recombinant CbpF (A) or strain 2B3 (B). Plates were overlaid with N-Fc, CEA or CD33-Fc (negative control) at equimolar concentrations indicated on the X-axis. Receptor binding was detected by using the CEACAM polyclonal antibody A0115, and anti-rabbit alkaline phosphatase conjugated secondary antibody. Dose dependent binding of both N-Fc and CEA were observed whether using immobilised recombinant CbpF or 2B3 lysate. Data are means of triplicates within one experiment +/- SD.

Table S2. Fusobacterial isolate CEACAM binding screen.

ID Number	Source	Species	CC1-Fc binding (Western blot/dot blot)	CProoF Group (where sequenced)
2B1	Pericoronitis	F. sp.	-	
2B2	Pericoronitis	F. sp.	+	II
2B3	Pericoronitis	F. sp.	+	II
2B4	Pericoronitis	F. sp.	+	II
2B5	Pericoronitis	F. sp.	+	
2B6	Pericoronitis	F. sp.	-	
2B7	Pericoronitis	F. sp.	+	
2B8	Pericoronitis	F. sp.	+	II
2B9	Pericoronitis	F. sp.	+	II
2B10	Pericoronitis	F. sp.	+	
2B11	Pericoronitis	F. sp.	+	
2B12	Pus	F. sp.	-	
2B13	Pus	F. sp.	+	
2B14	Pus	F. sp.	-	
2B15	Pus	F. sp.	+	
2B16	Periodontal pocket	F. sp.	+	II
2B17	Periodontal pocket	F. sp.	+	I
2B18	Periodontal pocket	F. sp.	-	
2B19	Periodontal pocket	F. sp.	-	
2B20	Periodontal pocket	F. sp.	-	
2B21	Periodontal pocket	F. sp.	+	
2B22	Periodontal pocket	F. sp.	+	
2B23	Periodontal pocket	F. sp.	+	I
2B24	Periodontal pocket	F. sp.	-	
2B25	Periodontal pocket	F. sp.	-	
2B26	Periodontal pocket	F. sp.	-	
2B27	Periodontal pocket	F. sp.	-	
2B28	Periodontal pocket	F. sp.	-	
2B29	ATCC25586 Cervico-facial lesion	F. nucleatum	+	I
2B30	Pus	F. nucleatum	-	
2B31	Pus	F sp.	-	
2B32	Periodontal pocket	F. sp.	+	
2B33	NCTC11326 Sinusitis upper jaw	F. vincentiii (formerly F. nucleatum sp. Fusiforme)	+	
2B34	Subgingival plaque	F. sp.	+	
2B35	Pus	F sp.	-	
2B36	ATCC33693 Periodontitis	F. periodonticum	-	
2B37	NCTC12276	F. animalis	-	
2B38	NCTC10562 Inflammed gingiva	F. polymorphum	-	
2B39	ATCC49256 Periodontal pocket	F. vincentii	+	I

Table S3. Primers used for *cbpf* sequencing and cloning

Primer	Sequence 5'-3'
CbpF For1	AAAAAATTTGTTAGTTTAAAATTAATTGTT
CbpF Rev1	CTATTTATTTTTTAATAACATATTTAA
CbpF For2	GAGTTTAGCTTTTGGATTTC
CbpF Rev2	TTGTACAGGAGCTTTTGGGTC
CbpF For3	GGAACAGGAGCTAATAGTATAGTAGCA
CbpF For4	GACCCAAAAGCTCCTGTACAAG
CbpF For5	AGGAGATATACCATGTCTTATTCAGCTGCACCAGTTATT
CbpF Rev5	GTGATGGTGATGTTTACCAGTGCCAAGCTTAGCTT
CbpF For6	AGGAGATATACCATGGCCCCAGCATTTGGAACA
CbpF Rev6	GTGATGGTGATGTTTAGCAGAACCTCCCCCTGT

Underlined sequences represent region of homology for pOPINE.

Table S4. Primers used for CEACAM mutant CEACAM-Fc constructs

Primer Sets (5' – 3')	Used to Create
<u>CTTGTCACGAATTCGATA</u>GGGCACCTCTCAGCCCCA<br><u>GTGAGTTTTGTCAGATCT</u>AGTGACTATGATCGTCTTGAC	CC1-3-Fc
<u>CAATCTGCCCCAGCAACTT</u>GGTGGCTACAGCTGG<br><u>AAGTTGCTGGGGCAGATTG</u>TGGACAAGGAG	F29G
<u>CAATCTGCCCCAGCAACTT</u>ATTGGCTACAGCTGG<br><u>AAGTTGCTGGGGCAGATTG</u>TGGACAAGGAG	F29I
<u>CAATCTGCCCCAGCAACTT</u>CGTGGCTACAGCTGG<br><u>AAGTTGCTGGGGCAGATTG</u>TGGACAAGGAG	F29R
<u>CAATCTGCCCCAGCAACTT</u>TATGGCTACAGCTGG<br><u>AAGTTGCTGGGGCAGATTG</u>TGGACAAGGAG	F29Y
<u>GAGTGGATGGCAACCGT</u>GAAATTGTAGGATATGC<br><u>ACGGTTGCCATCCACTC</u>TTTCCCCTTTG	Q44E
<u>GAGTGGATGGCAACCGT</u>CTAATTGTAGGATATGC<br><u>ACGGTTGCCATCCACTC</u>TTTCCCCTTTG	Q44L
<u>GAGTGGATGGCAACCGT</u>CGAATTGTAGGATATGC<br><u>ACGGTTGCCATCCACTC</u>TTTCCCCTTTG	Q44R
